# CSF1R inhibition rescues tau pathology and neurodegeneration in an A/T/N model with combined AD pathologies, while preserving plaque associated microglia

**DOI:** 10.1186/s40478-021-01204-8

**Published:** 2021-06-08

**Authors:** Chritica Lodder, Isabelle Scheyltjens, Ilie Cosmin Stancu, Pablo Botella Lucena, Manuel Gutiérrez de Ravé, Sarah Vanherle, Tim Vanmierlo, Niels Cremers, Hannah Vanrusselt, Bert Brône, Bernard Hanseeuw, Jean-Noël Octave, Astrid Bottelbergs, Kiavash Movahedi, Ilse Dewachter

**Affiliations:** 1 Department of Neurosciences, Biomedical Research Institute, Hasselt University, Hasselt, Belgium; 2 Myeloid Cell Immunology Lab, VIB Center for Inflammation Research, Brussels, Belgium; 3 Lab of Cellular and Molecular Immunology, Vrije Universiteit Brussel, Brussels, Belgium; 4 Institute of Neuroscience, Université Catholique de Louvain, Brussels, Belgium; 5 Department of Neurology, Cliniques Universitaires Saint-Luc, Brussels, Belgium; 6 Gordon Center for Medical Imaging, Department of Radiology, Massachusetts General Hospital, Harvard Medical School, Boston, MA USA; 7 Neuroscience Department, Janssen Research and Development, A Division of Janssen Pharmaceutica NV, Beerse, Belgium; 8 Laboratory for Molecular and Cellular Therapy, Department of Biomedical Sciences, Vrije Universiteit Brussel, Beerse, Belgium

**Keywords:** Alzheimer’s disease, Amyloid pathology, Tau pathology, Neurodegeneration, ATN-continuum, Microgliosis, Microglial profiling, CSF1R inhibition

## Abstract

Alzheimer's disease (AD) is characterized by a sequential progression of amyloid plaques (A), neurofibrillary tangles (T) and neurodegeneration (N), constituting ATN pathology. While microglia are considered key contributors to AD pathogenesis, their contribution in the combined presence of ATN pathologies remains incompletely understood. As sensors of the brain microenvironment, microglial phenotypes and contributions are importantly defined by the pathologies in the brain, indicating the need for their analysis in preclinical models that recapitulate combined ATN pathologies, besides their role in A and T models only. Here, we report a new tau-seed model in which amyloid pathology facilitates bilateral tau propagation associated with brain atrophy, thereby recapitulating robust ATN pathology. Single-cell RNA sequencing revealed that ATN pathology exacerbated microglial activation towards disease-associated microglia states, with a significant upregulation of *Apoe* as compared to amyloid-only models (A). Importantly, Colony-Stimulating Factor 1 Receptor inhibition preferentially eliminated non-plaque-associated versus plaque associated microglia. The preferential depletion of non-plaque-associated microglia significantly attenuated tau pathology and neuronal atrophy, indicating their detrimental role during ATN progression. Together, our data reveal the intricacies of microglial activation and their contributions to pathology in a model that recapitulates the combined ATN pathologies of AD. Our data may provide a basis for microglia-targeting therapies selectively targeting detrimental microglial populations, while conserving protective populations.

**Supplementary Information:**

The online version contains supplementary material available at 10.1186/s40478-021-01204-8.

## Introduction

Brains of AD patients are diagnostically characterized by amyloid plaques (A), neurofibrillary tangles (T) and neurodegeneration (N), which develop in a characteristic spatiotemporal way [24, 47]. Amyloid pathology can precede AD-symptoms up to a decade in time, while progressive tau pathology closely correlates with symptom progression and neurodegeneration [19, 27, 28]. To develop a biological definition of AD, biomarkers are used as proxies for the respective pathological changes in the brain, as framed in the ATN classification (NIA-AA framework) [27, 28]. Within this framework, different pathological stages of AD are presented, which range from an amyloid-stage to those that include progressive tau pathology and neurodegeneration. While the different pathological stages of the ATN continuum are well-defined, their interrelation, interaction and synergisms remain incompletely understood, yet hold key insights for therapeutic design. A better understanding of these synergisms and its modulation by neuroinflammation requires detailed analysis in humans, but also in preclinical models that recapitulate progressive combined ATN pathology, compared to models with A and T pathology only.

Mounting evidence of genetic studies and pathological analyses have convincingly implicated inflammation in AD and tauopathies in general [22, 31, 47, 48, 52]. Within the genetic risk factors associated with AD, many genes are expressed in microglia or macrophages [31, 52]. Furthermore, using pathological and imaging analysis, microgliosis has been invariably associated with both amyloid and tau pathology [3, 5, 9, 26, 47]. The advent of single-cell RNA sequencing (scRNA-Seq) has started to reveal the nature of microglial responses to AD pathology at single cell level. Seminal studies have shown that in mouse models of amyloid pathology, a subset of microglia transition towards a disease-associated state, which is characterized by the induction of phagocytosis and lipid metabolism genes [29, 30, 65]. The transcriptional response in these disease-associated microglia or DAM is at least partly induced via an APOE-TREM2-mediated signaling pathway [29, 30]. Importantly, both APOE and TREM2 are key genetic risk factors for human AD. However, initial studies that have relied on single-nucleus RNA sequencing (snRNA-Seq) to profile brains from AD patients, were unable to confirm the core mouse DAM signature as a response to AD pathology in human microglia [18, 40, 43, 74]. This could point towards species-related differences. Alternatively, it may result from a shortcoming of the snRNA-Seq approach, which may fail to detect many activation genes in microglia [62]. In this respect it must be noted that in a recent study, DAM-like microglia have been identified in human AD brains [12], using a microglial enrichment strategy. Despite the current complexity in the field, snRNA-Seq analysis did consistently reveal the upregulation of APOE and TREM2, both risk genes in AD, in human AD microglia, similar to what is observed in mouse DAM [74]. Taken together, these data support a role for microglia in AD, while their exact role in the progression along the ATN axis requires further detailed analysis in preclinical models. As single-cell microglial profiling has currently only been performed under conditions of isolated amyloid or tau pathology in preclinical models, it remains to be investigated how microglia react in models that combine amyloid plaque formation with tau propagation and neuronal atrophy.

While microglia are clearly implicated in AD, an ongoing conundrum is whether they play a beneficial or detrimental role, or both. Preclinical studies that have employed tau models all point towards microglia as drivers of pathology [2, 16, 25, 31, 35, 38, 39, 56, 72]. In settings of isolated tauopathy, microglial depletion reduces disease severity [2, 31, 38]. Along the same line, TREM2 deficiency and inactivating TREM2 mutations were shown to attenuate neuroinflammation and to attenuate tau-mediated pathologies [16, 35] in tau only models. However, in the presence of amyloid pathology TREM2 deficiency and inactivating TREM2 mutations, increased plaque-associated tau-positive dystrophic neurites, an early form of tau pathology [34]. It is clear that microglial contribution to AD progression relies on the type of pathology (amyloid vs. tau) and on the disease stage (early to late). Studies that have investigated microglial contribution to amyloid pathology have yielded mixed conclusions, indicating detrimental and protective contributions. However, a clear line of evidence suggests that microglia are critical for the formation of amyloid plaques. Microglial depletion at early stages reduces plaque load [53, 54]. In its extreme form, large scale and sustained microglial depletion starting from an early age could completely impair plaque formation [54]. Instead, Aβ deposits were now observed in cortical blood vessels, which was reminiscent of cerebral amyloid angiopathy [54]. This highlights the central role of microglia for dealing with the accumulating amyloid load via the triggering of plaque formation. At later disease stages, when mature amyloid plaques are abundant, microglial depletion no longer affects plaque load [55]. However, microglia remain important for compacting plaques and limiting their toxicity [8, 68, 70, 73], a process in which TREM2 has been shown to play a key role [68, 70, 73]. As it is emerging that microglia may limit amyloid toxicity and formation of plaque-associated dystrophic neurites, but also exacerbate tauopathy, a remaining question is how these cells shape overall disease pathology in combined models that recapitulate the human ATN spectrum. In an amyloid-facilitated tau seed model [21], TREM2 deficiency reduces microgliosis around amyloid plaques resulting in an enhanced spreading of neuritic plaque tau aggregates [34]. This indicates the importance of plaque-associated microglia in the early phase of induction of tau pathology. However, the role of microglia in combined presence of amyloid pathology, neurofibrillary tangles and brain atrophy, remains to be further investigated, and strategies to specifically target detrimental microglia, while preserving activity of protective microglia, need to be developed.

Here we report the generation of a new model of amyloid facilitated tau-seeding, which results in strong tau pathology, primarily linked to the formation of neurofibrillary tangles. Importantly, mice exhibited severe hippocampal and cortical atrophy, thereby reproducing the combined ATN spectrum. By relying on scRNA-Seq combined with microglial depletion we analyzed microglial responses and contribution to pathology. This revealed that ATN pathology exacerbates microglial activation towards a DAM-like state, with a significant upregulation of *Apoe* as compared to amyloid-only models. Blocking CSF1R signaling via a small-molecule inhibitor differentially impacted plaque versus non-plaque associated microglia/DAMs, as revealed by scRNA-Seq analysis. This highlights how CSF1R inhibition can be used to selectively deplete specific microglial subsets. Importantly, this CSF1R-inhibition regimen significantly attenuated tau pathology and neuronal atrophy. Together, our data provide new insights that can form a basis for future treatment avenues for AD.

## Materials and methods

### Animals

We crossed the in-house bred and well-characterized strains of hemizygous 5xFAD mice overexpressing mutant human APP695 carrying EOFAD mutations K670N/M671L (Swedish), I716V (Florida), V717I (London) and mutant human PS1 harboring 2 EOFAD mutations (M146L and L286V) driven by the thymocyte differentiation antigen 1 (ThyI) promoter, generated by the group of R. Vassar [42] (F^+^ mice), and hemizygous tau P301S transgenic mice (PS19), expressing human Tau-P301S (1N4R) driven by the mouse prion protein promoter (T^+^ mice), generated by the group of V. Lee [72], to generate heterozygous F^+^/T^+^ mice and the different single parental lines and non-transgenic strains. Animals were housed under regular conditions in a temperature-controlled room (20 ± 3 °C) on a 12-h day-night light cycle and with access to food and water ad libitum. All experiments were approved by the ethical committee for animal welfare of Hasselt University.

### Tau seeding and stereotactic surgery

Tau seeds were generated as described previously [58]. Tau fragments (tauP301L tau) containing the four repeat domain [K18; Q244-E373 (4RTau)], N-terminally Myc tagged were produced in Escherichia coli. Tau-PFFs (synthetic preformed fibrils) were obtained by incubation of tau fragments (66 µM) at 37 °C for 5 days in the presence of heparin (133 µM) in 100 mM ammonium acetate buffer (pH 7.0). Following centrifugation (100 000 g, 1 h, 4 °C) the pellet was resuspended in the same buffer (333 µM final) and sonicated before use. Tau fibrilization was confirmed using ThioT assay (ThioS, Sigma-Aldrich, St. Louis, MO, USA) and immunoblotting.

Mice were anaesthetized by intraperitoneal injection with a mixture of ketamine 10% w/v (Anesketin, Dechra), xylazine 2% w/v (Rompun, Bayer) and PBS (1 and 0.12 mg/10 g body weight ketamine and xylazine, respectively, dose volume 0.1 ml/10 g). After anaesthetizing the mice, PBS or sonicated pre-aggregated tau-PFFs (5µL; 333 µM) were unilaterally (right hemisphere) injected in to 4 months old mice. Stereotactic injections were performed in the hippocampal region (A/P − 2.0 mm; L + 1.4 mm; D/V − 1.4 mm, relative to bregma) and frontal cortex (A/P + 2.0 mm; L + 1.4 mm; D/V − 1.0 mm, relative to bregma), using a 10 µL Hamilton syringe at a speed of 1 µL per min. The needle was kept in place for an additional 5 min after injection.

### Behavioral analysis

The inverted grid hanging test was used to evaluate the ability of the mouse to grasp an elevated horizontal grid and remain suspended for 2 min. The animal was placed on the grid (40 cm × 20 cm/0.5 cm meshes) and positioned 50 cm above a flat, soft surface. The latency for the animal to drop off was then measured (in seconds).

### Immunohistological analysis

Three months after injection, animals were transcardially perfused with ice cold phosphate-buffered saline (PBS) for 2 min. The brains were dissected and immersion fixed in 4% paraformaldehyde (PFA) in PBS for 24 h at 4 °C for histological analyses. Sagittal sections of 40 µm were cut on a vibrating HM650V microtome (Thermo Fisher Scientific, Waltham, MA, USA). Immunohistochemistry was performed on free-floating sections with incubation of anti-tau P-S202/T205 (AT8; Thermo Fisher Scientific, Waltham, MA, USA), anti-Iba1 (Fujifilm Wako, Neuss, Germany), anti-neuronal nuclei (NeuN; Merck Millipore, Burlington, MA, USA) and anti-Aβ (W02; Invitrogen, Carlsbad, CA, USA) anti-bodies. The slices were then incubated with the appropriate AlexaFluor-488, AlexaFluor-568, and AlexaFluor-647 coupled secondary antibodies (Invitrogen). Staining with Thioflavin S (ThioS, Sigma-Aldrich, St. Louis, MO, USA) and Gallyas silver staining (all chemicals from Sigma-Aldrich) were performed as previously described [56], and are used to demonstrate mature NFTs by binding to β-sheet structures. Images were acquired with a Leica DM400 B LED fluorescence microscope (Leica, Diegem, Belgium), silver staining was assessed using a bright-field microscope. All images were analyzed using ImageJ open-source software (National Institutes of Health, Bethesda, MD, USA). Quantitative analysis of tau pathology was performed on AT8 stained vibratome sections. Well-defined sagittal sections at 1.32 mm lateral from bregma were selected for quantification of AT8 positive pathology. Tau pathology was analyzed by measuring the area occupied by tau tangles relative to the total image area of the brain regions of interest, using Image J software (U.S. National Institutes of Health, Bethesda, MD, USA). Amyloid-pathology and microgliosis were analyzed in a similar fashion by measuring the W02 and Iba1 positive area, respectively. The extent of amyloid-pathology, tau pathology and microglia in the cortex were analyzed by measuring the cortex, and measuring the positively stained W02, AT8 or Iba1 area % relative to the total cortex area. Hippocampal and cortical surface area were measured by delineating the brain structures as defined by the Mouse brain atlas and measuring the structure’s absolute surface area in µm^2^ on 5 × digital images using Image J software. Hippocampal volume was measured by tracing the hippocampal surface every sixth sagittal section, resulting in 240 µm between each analyzed section. Hippocampal volume was calculated using the following formula: volume = (sum of area) * 0.24 mm. Quantification started at 3.72 mm and ended at 0.48 lateral from bregma. Plaque-associated microglia were measured as the Iba1 positive area colocalizing with and directly bordering W02 positive area. Generation of heat maps of tau pathology was performed on sagittal sections. In sagittal brain sections at 1.32 mm lateral from bregma, tau pathology was semi-quantitatively analyzed per region based on the Mouse brain atlas. The extent of AT8 positive tau pathology was scored as 0–3 (with 0; no tau pathology and 3; extensive tau pathology) in tau-seeded and non-seeded F^−^/T^+^ and F^+^/T^+^ mice (n = 8; n = 6; n = 9; n = 9). Average scores from these mice were assigned colors accordingly and were filled in on a map based on the Mouse brain atlas to represent extent and distribution of tau pathology.

### Brain single cell isolation and single-cell RNA sequencing

Seven-month-old mice, 4 mice per condition (4 WT control mice (F^−^/T^−^), 4 5xFAD mice (F^+^/T^−^) and 4 tau-seeded 5xFAD/PS19 tau transgenic mice (F^+^/T^+^) 3 months post injection), were sacrificed for brain isolation and single-cell processing. For single cell experiments related to PLX3397 treatment, 2 mice per condition were used (2 tau-seeded 5xFAD/PS19 tau transgenic mice (F^+^/T^+^) which received PLX3397 chow 1.5 months after tau-seeding for 1.5 months, and 2 tau-seeded 5xFAD/PS19 tau transgenic mice (F^+^/T^+^) which received control chow 1.5 months after tau-seeding for 1.5 months). All processing and tissue-collection was performed as previously described [65], using the Act-Seq method [71] to limit dissociation-induced gene expression. All steps were performed at 11 °C. In brief, the brain was extracted and placed in ice-cold RPMI (Gibco), containing 30 μM Actinomycin D (ActD) (Sigma, No. A1410). The brains of n = 4 F^−^/T^−^, and n = 4 F^+^/T^−^ mice were pooled, respectively. The F^+^/T^+^ brains were split per hemisphere: 4 hemispheres ipsilateral of the tau-seed injection were pooled and 4 hemispheres contralateral to the injection were pooled. For PLX3397 single cell experiments, the hemispheres were not split. The brains were cut into small pieces and incubated with enzyme mix (30 U ml^−1^ DNAse I (Roche), 10 U ml^−1^ collagenase type I (Worthington) and 400 U ml^−1^ collagenase type IV (Worthington) diluted in 1 × Hanks’ buffered salt solution (Gibco)), containing 15 μM ActD at 11 °C for 40 min. Every 10 min the solution was cut and resuspended to ensure full dissociation of the tissue. Subsequently, the solution was resuspended, filtered twice over a 100 μm nylon filter, using RPMI with 3 µM ActD and centrifuged. The pellet was resuspended in 5 ml 70% standard isotonic percoll (SIP, GE Healthcare) diluted in 1 × Hanks’ buffered salt solution and gently overlaid with 5 ml of 37% SIP, followed by a 5 ml layer of 30% SIP, forming a three-layered density gradient (centrifuged at 800 g, 4 °C, 30 min without acceleration/braking). All gradient buffers contained 3 µM ActD. The 70/37% interphase containing immune cells was collected, centrifuged and resuspended in fluorescent activated cell sorter (FACS) buffer (2 mM EDTA (Duchefa), 2% heat-inactivated fetal calf serum (Gibco) dissolved in 1 × Hanks’ buffered salt solution), containing 3 µM ActD. The dissociation process required 4 h. Following single-cell isolation, cells were blocked with rat anti-mouse CD16/CD32 (clone 2.4G2) for 15 min on ice. Subsequently, the cells were stained with anti-CD45-APCCy7 (30-F11, BioLegend) for 20 min on ice and washed. All CD45^+^ immune cells were sorted in ME-medium (RMPI medium supplemented with 20% heat-inactivated fetal calf serum (Gibco), 300 μg ml^−1^ l-glutamine (Gibco), 100 units ml^–1^ penicillin and 100 μg ml^–1^ streptomycin (Gibco), 1 mM non-essential amino acids (Gibco), 1 mM sodium pyruvate (Gibco) and 0.05 mM 2-mercaptoethanol (Sigma)), containing 3 µM ActD, using a BD FACS ARIA II, or FACS ARIA III, with a sorting nozzle of 85 µm. DAPI (Sigma) or 7-AAD (BioLegend) was used to exclude dead cells; cell viability before and after cell sorting exceeded 90%. Sorted cells were centrifuged at 4 °C at 400 g, then resuspended in PBS + 0.04% bovine serum albumin at room temperature to yield an estimated final concentration of 1000 cells μl^–1^.

Cellular suspensions were loaded on a Chromium Chip B (10 × Genomics, No.1000074), or Chip G (10 × Genomics, No. 2000177) on a GemCode Single Cell Instrument (10 × Genomics) to generate single-cell gel beads-in-emulsion (GEM). GEMs and scRNA-Seq libraries were prepared using the GemCode Single Cell 3’ Gel Bead and Library Kit (v3 and v3.1, 10xGenomics, No. 1000075) and the Chromium i7 Multiplex Kit (10 × Genomics, No. 120262) according to the manufacturer’s instructions. Briefly, GEM reverse-transcription incubation was performed in a 96-deep-well reaction module at 53 °C for 45 min, 85 °C for 5 min and ending at 4 °C. Next, GEMs were broken and complementary DNA (cDNA) was cleaned up with DynaBeads MyOne Silane Beads (10 × Genomics, No. 2000048) and SPRIselect Reagent Kit (Beckman Coulter, No. B23318). Full-length, barcoded cDNA was PCR amplified with a 96-deep-well reaction module at 98 °C for 3 min, eleven cycles at 98 °C for 15 s, 63 °C for 20 s and 72 °C for 1 min, followed by one cycle at 72 °C for 1 min and ending at 4 °C. Following cleaning up with the SPRIselect Reagent Kit and enzymatic fragmentation, library construction to generate Illumina-ready sequencing libraries was performed by the addition of R1 (read 1 primer), P5, P7, i7 sample index and R2 (read 2 primer sequence) via end-repair, A-tailing, adapter ligation, post-ligation SPRIselect cleanup/size selection and sample index PCR. The cDNA content of pre-fragmentation and post-sample index PCR samples was analyzed using the 2100 BioAnalyzer (Agilent).

Sequencing libraries were loaded on an Illumina HiSeq4000 or Illumina NovaSeq6000 flow cell with sequencing settings following the recommendations of 10 × Genomics (Read 1: 28 cycles, i7 Index: 8 cycles, i5 Index: 0 cycles, Read 2: 91 cycles, 2.73 nM loading concentration). The Cell Ranger pipeline (10 × Genomics) was used to perform sample demultiplexing and to generate FASTQ files for read 0, read 2 and the i7 sample index. Read 2, containing the cDNA, was mapped to the reference genome (mouse mm10) using STAR. Subsequent barcode processing, unique molecular identifiers filtering and single-cell 3’ gene counting was performed using the Cell Ranger suite and Seurat v.3.0.1. The total number of cells across all libraries was 19,252 cells. The average of the mean reads per cell across all libraries was 69,801, with an average sequencing saturation of 76,05%, as calculated by Cell Ranger. Digital gene expression matrices were preprocessed and filtered using the Scater R packages [37]. Outlier cells were first identified based on three metrics (library size, number of expressed genes and mitochondrial proportion); cells were tagged as outliers when they were four median absolute deviations distant from the median value of each metric across all cells. Secondly, a principal component analysis plot was generated based on multiple metrics: ‘pct_counts_in_top_100_features’, ‘total_features_by_counts’, ‘pct_counts_feature_control’, ‘total_features_by_counts_feature_control’, ‘log10_total_counts_endogenous’ and ‘log10_total_counts_feature_control’. Outlier cells in this principal component analysis plot were identified using the R package mvoutlier. Low-abundance genes were removed using the ‘calcAverage’ function and the proposed workflow. By means of the seuratMerge function, a merge was created of the raw counts of the F^−^/T^−^, F^+^/T^−^, F^+^/T^+^ ipsilateral hemispheres (ipsi) and F^+^/T^+^ contralateral hemispheres (contra), and another merge was created of the F^+^/T^+^ which received PLX3397 and the F^+^/T^+^ which did not receive PLX3397. The two resulting datasets were normalized in Seurat by a global-scaling normalization and log-transform method ‘LogNormalize’ that normalizes the gene expression measurements for each cell by the total expression, and multiplies it by a scale factor (10,000), and log-transforms the result. Highly variable genes were detected in Seurat according to the method described in Stuart et al. [60] and the data was scaled by linear transformation. Subsequently, the highly variable genes were used for unsupervised dimensionality reduction techniques and principal component analysis. Unsupervised clustering of the cells was performed using graph-based clustering based on SNN-Cliq and PhenoGraph as implemented in the Seurat v.3.0.1 R package (default parameters). Clustering was visualized in two-dimensional scatter plots (via UMAP) using the Seurat v.3.0.1 package. All scRNA-seq data are deposited at GEO (NCBI) with accession code GSE176032.

### PLX3397 treatment

For the elimination of microglia, we used PLX3397 (Adooq Bioscience, Irvine, CA, USA) a well characterized CSF1R inhibitor. PLX3397 was blended with mouse chow (V1524-000 Ssniff) to a concentration of 1000 mg/kg PLX3397 chow. Mice were randomly assigned PLX3397 diet or control diet and were treated for 1.5 months starting at 1.5 month post injection.

### Statistical analysis

Data were statistically analyzed using GraphPad Prism version 9.0 (GraphPad Software Inc, San Diego, USA). Normal distribution was tested using Shapiro–Wilk test. Data were analyzed using unpaired t-test, two-way analysis of variance (ANOVA) with Tukey’s test for multiple comparison, one-way ANOVA with Dunnett’s multiple comparison test or Tukey’s multiple comparison test for normally distributed data, or Kruskal–Wallis test with Dunn’s multiple comparison test for non-normally distributed data. Results were presented as mean ± standard error (SEM). Correlation was measured using Pearson’s correlation analysis. A probability of *p* < 0.05 was considered significant. *p < 0.05, **p < 0.01, ***p < 0.001, ****p < 0.0001. Differential gene expression was assessed using the Wilcoxon rank sum test (two-sided) as implemented in the Seurat R package. *p* value adjustment was performed using Bonferroni correction based on the total number of genes in the dataset.

## Results

### Tau-seeding in 5xFAD/PS19 mice results in a bilateral propagation of tau pathology associated with neuronal atrophy, recapitulating the ATN pathological features of human AD

**Fig. 1:**
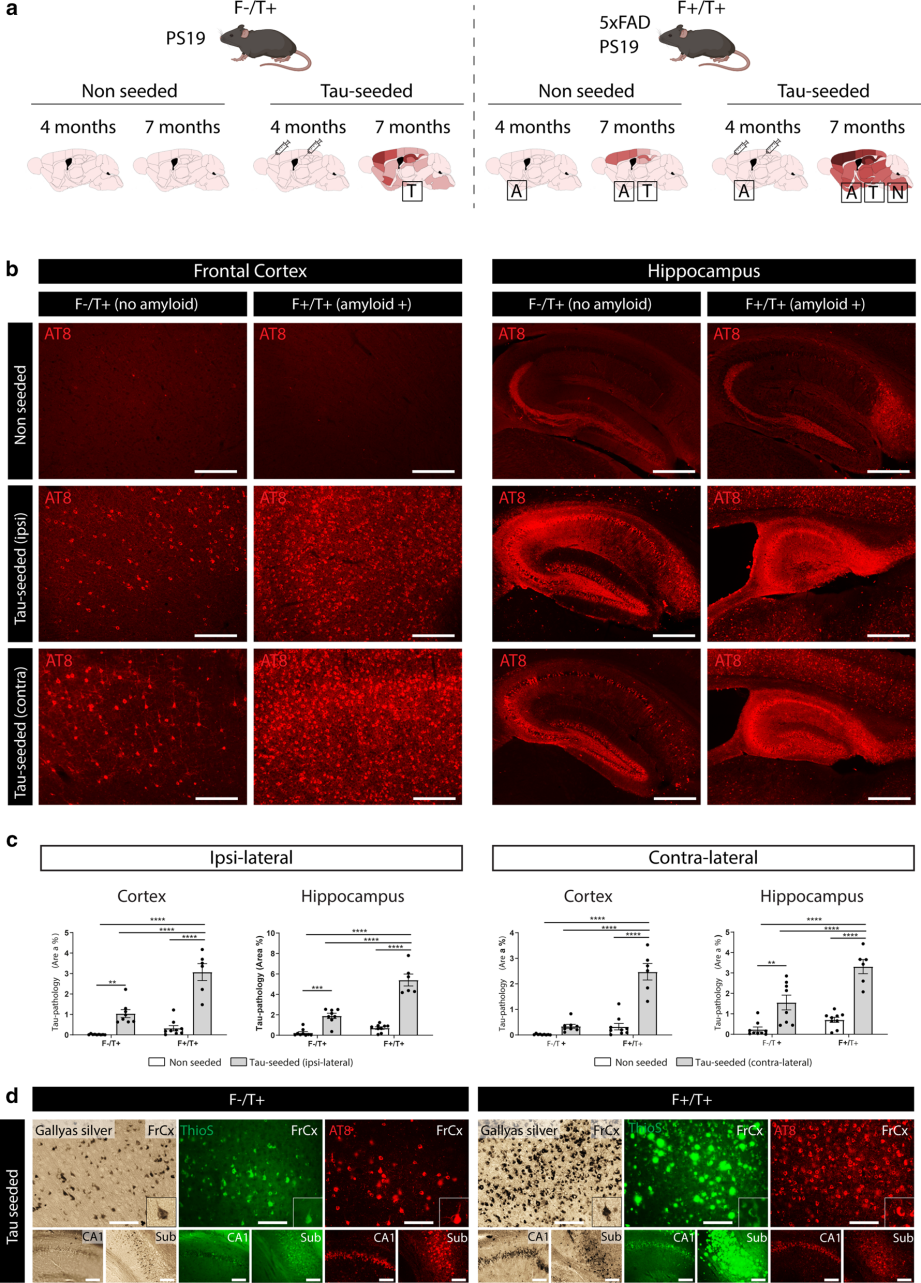
Amyloid pathology aggravates tau-seeded tau pathology and propagation. **a** Schematic overview of the tau-seed model. Tau-seeding is performed at 4 months of age in F^−^/T^+^ (PS19) or F^+^/T^+^ (5xFAD/PS19) mice. Pathological analysis occurs at 7 months of age. **b** Representative images of the ipsi- and contralateral frontal cortex (scale bar = 250 µm) and hippocampus (scale bar = 500 µm) of F^−^/T^+^ and F^+^/T^+^ mice following tau-seeding, and their non-seeded controls at 7 months (3 months post-injection), immunohistochemically stained with anti-phospho-tau (pSer202/Thr205) antibody AT8. **c** Quantitative analysis of AT8 signal in the ipsi- and contralateral cortex and hippocampus of tau-seeded F^−^/T^+^ (n = 8) and F^+^/T^+^ mice (n = 6) compared to non-seeded F^−^/T^+^ and F^+^/T^+^ mice (n = 9, n = 9). Two-way ANOVA, Tukey’s test for multiple comparison. Data are presented as mean ± SEM; **p < 0.01; ***p < 0.001; ****p < 0.0001 **d** Gallyas Silver and Thioflavin S (ThioS) staining concurred with AT8 staining, indicating formation of mature NFTs. ThioS, binding β-sheet structures, and silver staining, both stain Aβ pathology and tau pathology concomitantly, preventing quantitative analysis of tau pathology only. These stains are presented to demonstrate the presence of aggregated tau. Scale bar = 100 µm. (FrCx = frontal cortex; CA1 = cornu ammonis 1; Sub = subiculum)

Previous seminal work has shown that injection of human AD-tau in mice that harbor amyloid plaques results in the propagation of endogenous tau aggregates that surround Aβ plaques (NP tau) or develop into neurofibrillary tangles (NFTs) [21, 66]. While this approach elegantly models amyloid-facilitated propagation of tau aggregates and provides key insights into the link between amyloid and tau pathology, no progression to neuronal atrophy was observed. Therefore, we aimed to generate a tau-seed model that captures the complete ATN spectrum, including robust neurodegeneration. Hereto we used crosses of PS19 mice (F^−^/T^+^) and 5xFAD (F^+^/T^−^) transgenic mice. PS19 transgenic mice display age-dependent development of tau pathology, starting from 11.5 months onwards, associated with a progressive neurodegenerative phenotype, including development of progressive motoric problems, clasping, hunchback development and premature death [58, 72]. 5xFAD transgenic mice develop robust amyloid pathology starting at the age of 2.5 months onwards in subiculum and subsequently extending in cortical and other brain regions, providing an excellent model to study Aβ-related pathology [42]. We assessed the consequences of tau-seeding in PS19 mice (F^−^/T^+^) and in 5xFAD/PS19 double transgenic animals (F^+^/T^+^) in which overexpression of human tau is combined with amyloid pathology. Intracerebral injections of pre-aggregated tau fragments (tau seeds) were performed at four months of age (Fig. 1a), when amyloid plaques are abundant in the cortex and hippocampus (Additional file 1: Supplementary Fig. 1). Three months post injection, tau pathology was assessed via immunostaining with the anti-phospho-tau (Ser 202, Thr205) antibody AT8. As we have shown previously [44, 58], tau-seeding significantly increased tau pathology in the cortex and hippocampus of PS19 mice, both in the ipsilateral and contralateral hemispheres (Fig. 1b, c). This confirms that tau seeds efficiently shorten the long lag-phase of tau aggregation. Importantly, tau-seeded F^+^/T^+^ mice exhibited significantly higher AT8 staining as compared to seeded F^−^/T^+^ mice (Fig. 1b, c, Additional file 3: Supplementary Fig. 3a,b), indicating that the presence of amyloid plaques strongly enhanced tau pathology. While AT8 pathology was strongly increased in F^+^/T^+^ brains, the AT8 staining patterns were comparable in both strains. Indeed, we did not observe NP tau in seeded F^+^/T^+^ mice. Furthermore, AT8 staining clearly correlated with Gallyas silver and Thioflavin S (Fig. 1d), which is indicative of mature NFTs. Mapping of AT8 staining showed a more extensive spreading of tau pathology in tau-seeded F^+^/T^+^ mice, both in the ipsi- and contralateral hemispheres and in brain regions distal from the initial injection site (Additional file 2: Supplementary Fig. 2a), including the brain stem and thalamus (Additional file 2: Supplementary Fig. 2b).

**Fig. 2:**
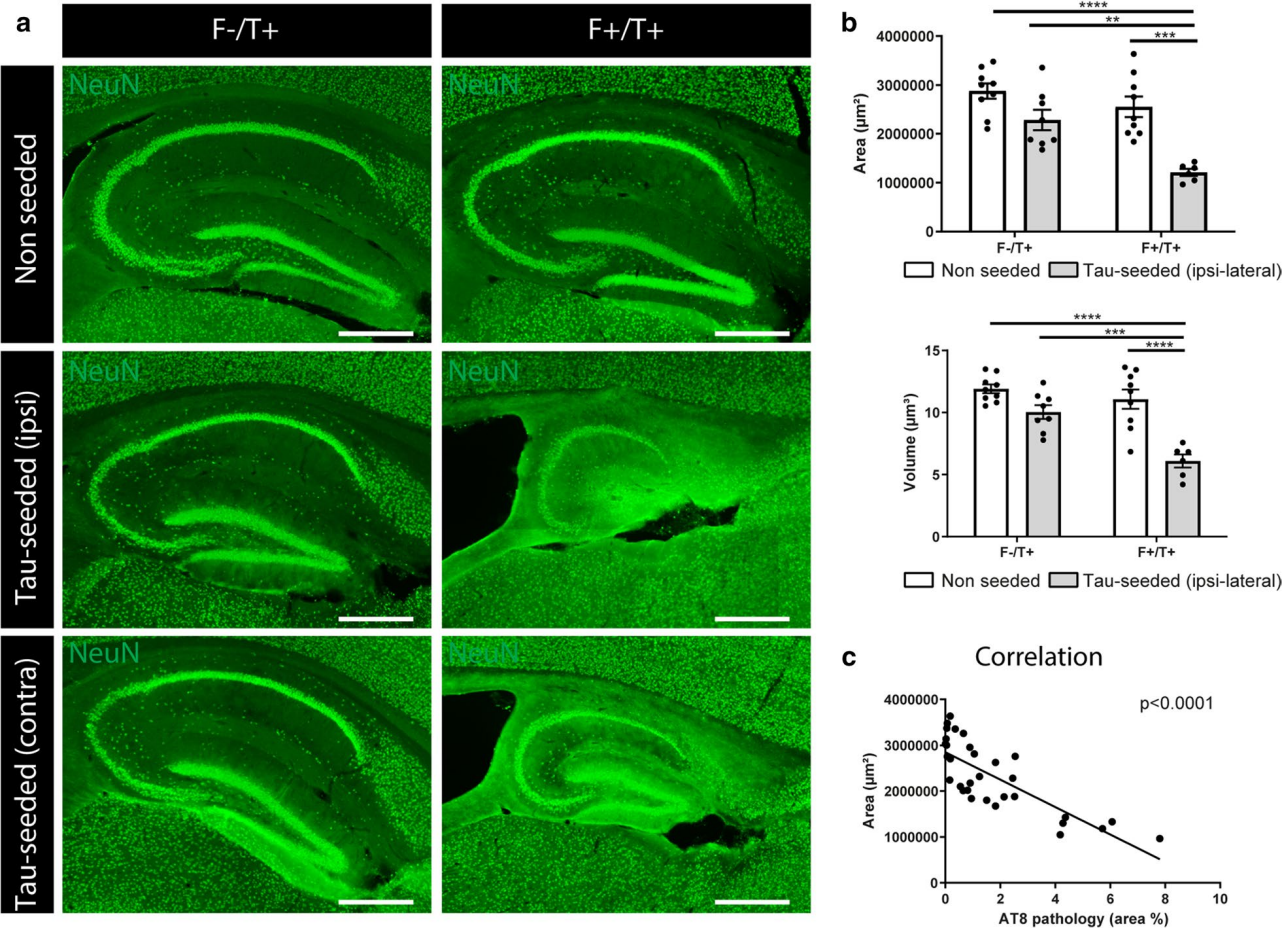
Amyloid pathology aggravates tau-induced atrophy. **a** Representative images of the hippocampus of tau-seeded F^−^/T^+^ and F^+^/T^+^ mice and their non-seeded littermates at 7 months (3 months post-injection), immunohistochemically stained with anti-NeuN antibody. Scale bar = 500 µm. **b** Quantification of hippocampal area and hippocampal volume of tau-seeded F^+^/T^+^ mice (n = 6) compared to tau-seeded F^−^/T^+^ mice (n = 8) and non-seeded F^−^/T^+^ and F^+^/T^+^ mice (area: n = 9; n = 9). Two-way ANOVA, Tukey’s test for multiple comparison. Data are presented as mean ± SEM; **p < 0.01; ***p < 0.001; ****p < 0.0001 **c** Correlation analysis between tau pathology in the hippocampus and hippocampal atrophy in 7 months old tau-seeded and non-seeded F^−^/T^+^ and F^+^/T^+^ mice. Pearson’s correlation analysis

To assess for neuronal atrophy, we performed a quantitative analysis of the hippocampal and cortical areas following NeuN staining. Tau-injected F^−^/T^+^ mice did not display significant hippocampal (Fig. 2a, b) or cortical (Additional file 4: Supplementary Fig. 4a,b) shrinkage as compared to non-seeded F^−^/T^+^ or F^+^/T^+^ littermates at 7 months of age. In contrast, strong hippocampal and cortical atrophy was observed in tau-seeded F^+^/T^+^ mice (Fig. 2a, b, Additional file 4: Supplementary Fig. 4a,b). Hippocampal shrinkage was macroscopically observable, and quantification indicated a significant reduction in hippocampal area and volume (Fig. 2b). Cortical and hippocampal atrophy was also observed in the contralateral hemisphere (Additional file 4: Supplementary Fig. 4d). Pearson’s correlation analysis revealed a significant negative correlation between AT8 staining and hippocampal (Fig. 2c) or cortical (Additional file 4: Supplementary Fig. 4c) area, suggesting a clear association between tau pathology and neuronal atrophy. Finally, we analyzed whether the development of motor deficits, characteristic for PS19 mice, were accelerated in tau-seeded F^+^/T^+^ mice. Motor coordination and grip strength were measured using the inverted grid hanging test. While scoring of hind limb clasping did not reveal significant differences in clasping scores, the inverted grid hanging performance, a more sensitive test, displayed a significant impairment in tau-seeded F^+^/T^+^ mice as compared to F^−^/T^+^ and non-seeded littermates (Additional file 4: Supplementary Fig. 4e).

We conclude that tau-seeding in 5xFAD/PS19 double transgenic mice results in amyloid-facilitated seeding and propagation of tau pathology, associated with hippocampal and cortical atrophy. This model thereby recapitulates the cardinal ATN pathological features of human AD and is hereafter referred to as ATN model.

### Single-cell analysis reveals that ATN pathology exacerbates microglial activation with elevated expression of *Apoe* as compared to amyloid only models

**Fig. 3:**
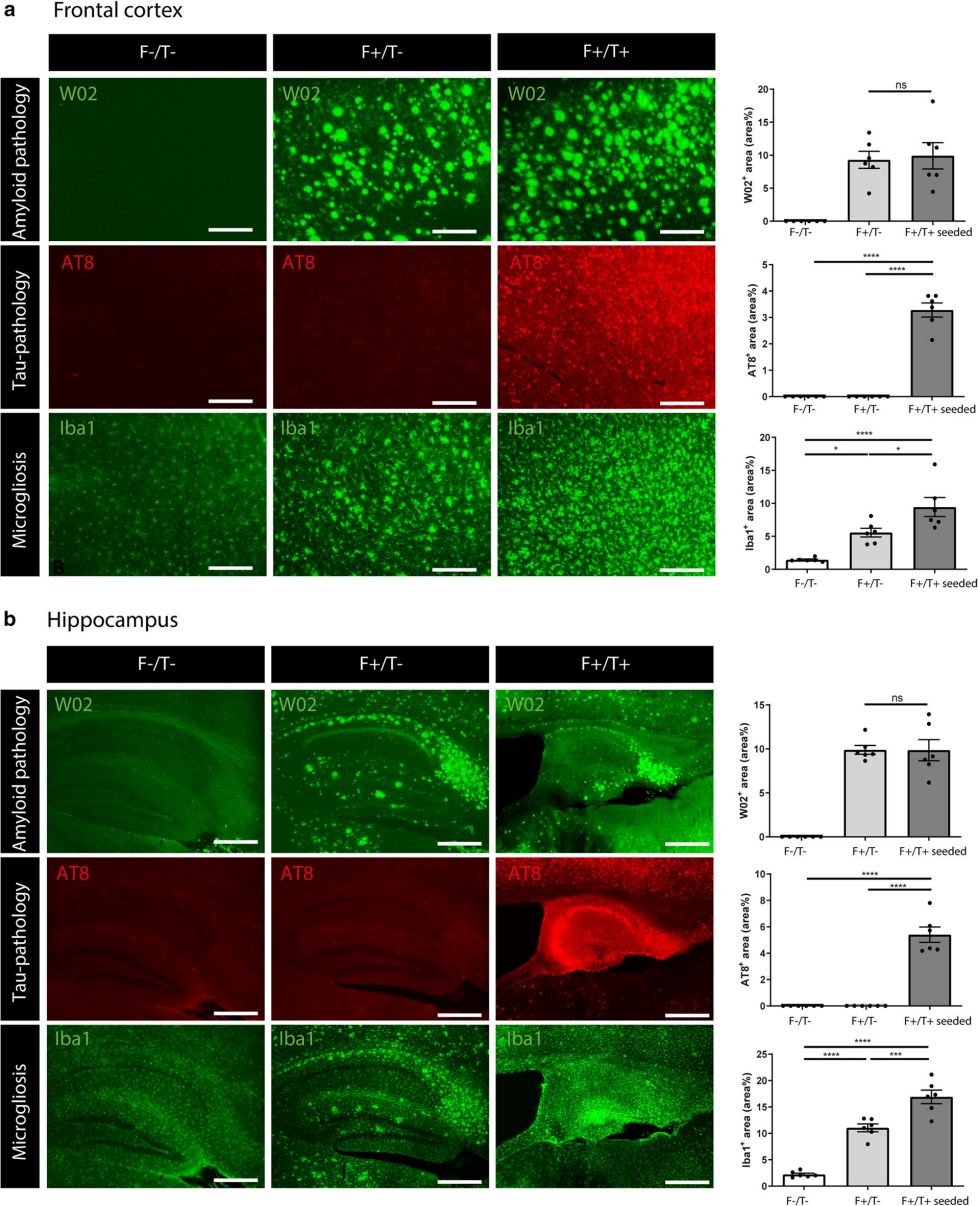
ATN pathology exacerbates microgliosis. **a**, **b** Representative images of (**a**) frontal cortex (Scale bar = 250 µm) and (**b**) hippocampus **(**Scale bar = 500 µm) of wildtype F^−^/T^−^, F^+^/T^−^ and tau-seeded F^+^/T^+^ mice at 7 months of age, immunohistochemically stained with anti-Aβ antibody W02, anti-phospho-tau (pSer202/Thr205) antibody AT8 and anti-Iba1 antibody. Quantitative analysis of W02, AT8 and Iba1 signal in F^−^/T^−^ (n = 6), F^+^/T^−^ (n = 6) and tau-seeded F^+^/T^+^ (n = 6) mice. One-way ANOVA with Tukey’s multiple comparison test (normally distributed); Kruskal–Wallis test with Dunn’s multiple comparison (non-normally distributed). Data are presented as mean ± SEM; *p < 0.05; ***p < 0.001; ****p < 0.0001

Brains of AD patients are invariably characterized by microgliosis throughout the different stages of disease progression. Microglial activation is also observed in 5xFAD mice, where single-cell analysis has revealed the existence of DAMs, which are enriched around amyloid plaques [29]. We wondered whether microglial activation would be altered under conditions of amyloid-facilitated tau propagation and neuronal atrophy, which is more reminiscent for the sequential changes that are observed in AD patients. To assess how pathology affects microglial activation, we assessed Iba1 immunostaining in the frontal cortex and hippocampus in conditions of amyloid and tau pathology and combined ATN pathology. Both amyloid (Fig. 3a, b; Additional file 5: Supplementary Fig. 5a,b) and tau pathology (Additional file 5: Supplementary Fig. 5a,b) significantly increased microglial activation compared to control mice. We next analysed microglial activation in subsequent stages of the ATN continuum, by comparing control mice (F^−^/T^−^), 5xFAD mice (F^+^/T^−^), which exhibited only amyloid pathology, and tau-seeded 5xFAD/PS19 double transgenic mice (F^+^/T^+^), which exhibited both amyloid and tau pathology (Fig. 3a, b). This showed a significant increase in Iba1 staining intensity in tau-seeded F^+^/T^+^ as compared to F^+^/T^−^ mice, suggesting an increase of microglial activation under ATN conditions (Fig. 3a, b).

**Fig. 4:**
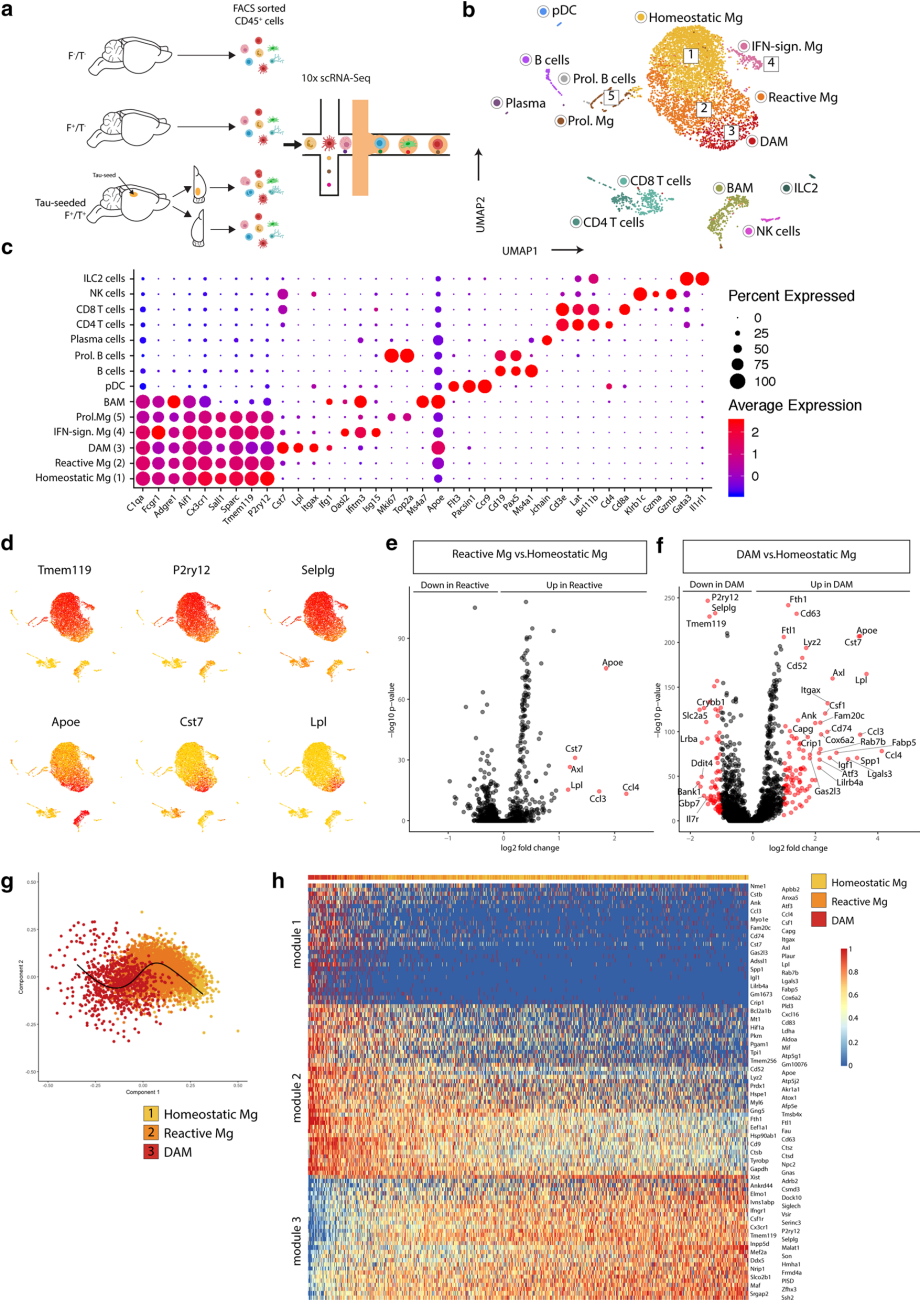
scRNA-Seq analysis of F^−^/T^−^, F^+^/T^−^ and tau-seeded F^+^/T^+^ brains reveals microglial activation and progression towards a DAM state. **a** Schematic overview of the 10 × chromium scRNA-Seq setup used on brains from F^−^/T^−^, F^+^/T^−^, and tau-seeded F^+^/T^+^ mouse models. Brains were collected from n = 4 mice for each condition. **b** UMAP-projection containing 842 F^−^/T^−^ cells, 2666 F^+^/T^−^ cells, and 1500 ipsilateral and 1500 contralateral tau-seeded F^+^/T^+^ cells which were randomly downsampled for visualization purposes in the UMAP plot. Clusters 1 to 5 correspond to different activation states of microglia populations. 1: homeostatic microglia, 2: reactive microglia, 3: DAM, 4: IFN-sign. microglia, 5: proliferative microglia. **c** Dot plot visualizing expression of key marker genes for each of the clusters that were identified in (**b**). **d** UMAP plots showing the expression of homeostatic microglia marker genes (top) and DAM marker genes (bottom). **e** Volcano plot showing genes that are DE between reactive microglia and homeostatic microglia. **f** Volcano plot showing genes that are DE between homeostatic microglia and DAM. DE threshold: -log_10_(adjusted *p*) > 5, log_2_(FC) > 1; *p* value adjustment was performed using Bonferroni correction (**e**–**f**). **g** SCORPIUS trajectory inference was run on microglia clusters 1, 2 and 3 from (**b**). Cells were automatically ordered along a linear trajectory and colored according to the cluster colors visualized in (**b**). **h** The top 100 genes that were found to define the trajectory in panel g, were clustered into three gene modules (normalized expression) that are down-or upregulated as homeostatic microglia transition towards DAM

**Fig. 5:**
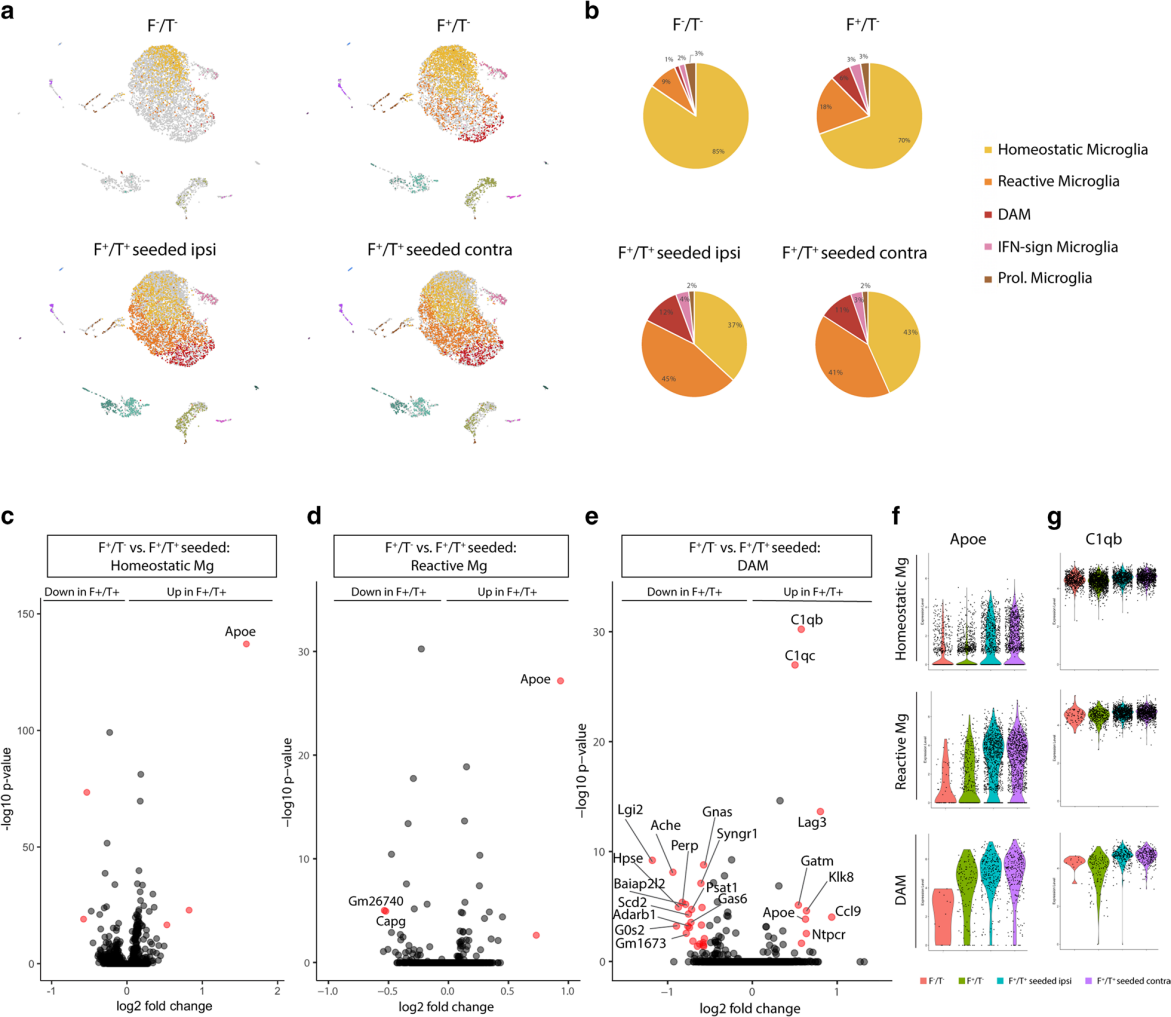
ATN conditions increase microglial transformation towards DAMs and result in a global upregulation of *Apoe*. **a** UMAP plots of the merged dataset of the F^−^/T^−^, F^+^/T^−^ whole brains and contra-and ipsilateral tau-seeded F^+^/T^+^ hemispheres as shown in Fig. 4b, where each UMAP plot highlights the cells from each condition separately. Clusters are colored according to the cluster colors shown in Fig. 4b. Only cells belonging to one of the 4 conditions are colored per panel, cells belonging to other conditions are depicted in grey. **b** Pie charts representing the percentages of the 5 distinct microglia subsets as identified in Fig. 4b, within total microglia, per condition. **c** Volcano plot showing genes that are DE in homeostatic microglia between F^+^/T^−^ and tau-seeded F^+^/T^+^ conditions. **d** Volcano plot showing genes that are DE in reactive microglia between F^+^/T^−^ and tau-seeded F^+^/T^+^ conditions. **e** Volcano plot showing genes that are DE in DAM between F^+^/T^−^ and tau-seeded F^+^/T^+^ conditions. DE threshold: − log_10_ (adjusted *p*) > 1.5, log_2_(FC) > 0.5; *p* value adjustment was performed using Bonferroni correction (**c**–**e**). **f** Violin plots showing the normalized gene expression of *Apoe* per cell in each of the 4 conditions for homeostatic microglia, reactive microglia and DAM. **g** Violin plots showing the normalized gene expression of *C1qb* per cell in each of the 4 conditions for homeostatic microglia, reactive microglia and DAM

To further characterize microglia at single-cell resolution, we performed scRNA-Seq analysis on CD45^+^ cells sorted from 7-month-old F^−^/T^−^, F^+^/T^−^ and tau-seeded F^+^/T^+^ mice, for which the tau-injected ipsi- and contralateral hemispheres were separately processed (Fig. 4a). This resulted in 842 captured cells from F^−^/T^−^ brains, 2666 cells from F^+^/T^−^ brains and 2966 and 4814 cells from the ipsi- and contralateral F^+^/T^+^ brains, respectively. The individual samples were combined in a single dataset on which we performed unsupervised clustering and dimensionality reduction via the Uniform Manifold Approximation and Projection (UMAP) technique (Fig. 4b). Immune cell clusters, which included microglia, border associated macrophages (BAMs), dendritic cells (DCs) and various lymphocyte subsets, were identified based on previously described gene expression signatures [65], for which a selection of genes are shown in Fig. 4c. Microglia were identified based on the expression of macrophage (e.g. *C1qa, Fcgr1, Aif1*) and microglial signature genes (*Sall1, Sparc, Tmem119*). We observed four main microglia subsets (clusters 1–4), in addition to a fraction of microglia that were actively proliferating (cluster 5) as indicated by the expression of cell cycle genes (*Mki67*, *Top2a*). Microglia in cluster 4 expressed interferon-induced genes (e.g. *Oasl2, Ifitm3, Isg15*) (Fig. 4c), suggestive of an interferon-mediated signaling response. Homeostatic microglia were contained within cluster 1, while microglia in clusters 2 and 3 exhibited a gradual reduction of homeostatic signature genes (e.g. *Tmem119, P2ry12, Selplg*) and an induction of DAM genes (e.g. *Apoe, Cst7, Lpl*) (Fig. 4d). This was further confirmed by differential expression analysis, revealing the upregulation of DAM genes in cluster 2 (Fig. 4e), which was further increased in cluster 3 (Fig. 4f). This indicates that cells in cluster 2 represent an intermediate state in between homeostatic microglia (cluster 1) and DAMs (cluster 3) and were termed reactive microglia. To better capture the gradual changes in microglial activation, we performed trajectory inference analysis on the homeostatic, reactive and DAM clusters using the Scorpius pipeline. This revealed a trajectory starting from homeostatic microglia and moving towards reactive microglia and subsequently DAMs (Fig. 4g). The most predictive genes that were up- or downregulated could be clustered across three gene modules (Fig. 4h), revealing genes that are induced (module 1), upregulated (module 2) or downregulated (module 3), as homeostatic microglia transform into DAMs (Fig. 4h). Importantly, the percentage of reactive microglia and DAMs were strongly increased in tau-seeded F^+^/T^+^ brains, totaling more than 50% of microglia (Fig. 5a, b). This indicates that ATN pathology results in a more global microglial activation as compared to amyloid only models. The percentages of proliferating and IFN-induced microglia were comparable across the different groups, suggesting that these microglial states were not AD-driven.

While the percentage of non-homeostatic microglia was increased in tau-seeded F^+^/T^+^ mice, we wondered whether the distinct microglial subsets would also exhibit model-specific transcriptional differences. Therefore, we compared the microglial subsets from tau-seeded F^+^/T^+^ mice with their counterparts from F^+^/T^−^ mice (Fig. 5c–e). Remarkably, this showed that for homeostatic and reactive microglia, the gene expression profiles were comparable across the two models, except for a significant upregulation of *Apoe* in tau-seeded F^+^/T^+^ mice (Fig. 5c, d). Both the expression level and percentage of *Apoe*-expressing cells was increased in cluster 1 and cluster 2 microglia from the ipsi- and contralateral hemispheres of tau-seeded F^+^/T^+^ brains (Fig. 5f). Even for DAMs in cluster 3, where *Apoe* levels were high, its expression was slightly increased in tau-seeded F^+^/T^+^ brains. These data were further corroborated by immunohistological analysis, demonstrating increased total ApoE staining, and ApoE staining in microglia, in tau-seeded F^+^/T^+^ brains compared to F^+^ brains (Additional file 6: Supplementary Fig. 6a–c). These DAMs also showed an increase of Complement component 1q genes as compared to their counterparts in F^+^/T^−^ mice (Fig. 5e, g).

We conclude that upon ATN pathology a higher percentage of microglia is driven towards an activated or DAM state. As amyloid pathology is comparable between F^+^/T^−^ mice and tau-seeded F^+^/T^+^ mice (Fig. 3), this suggests that under ATN pathology, some DAMs are not associated with amyloid plaques and likely arise in response to tau pathology and/or neurodegeneration [30]. While the transcriptional programs of reactive microglia or DAM were comparable between the amyloid-only or ATN model, the latter exhibited an upregulation of *Apoe* across all microglial clusters*.*

### CSF1R inhibition preferentially depletes non-plaque associated microglia during ATN pathology

**Fig. 6:**
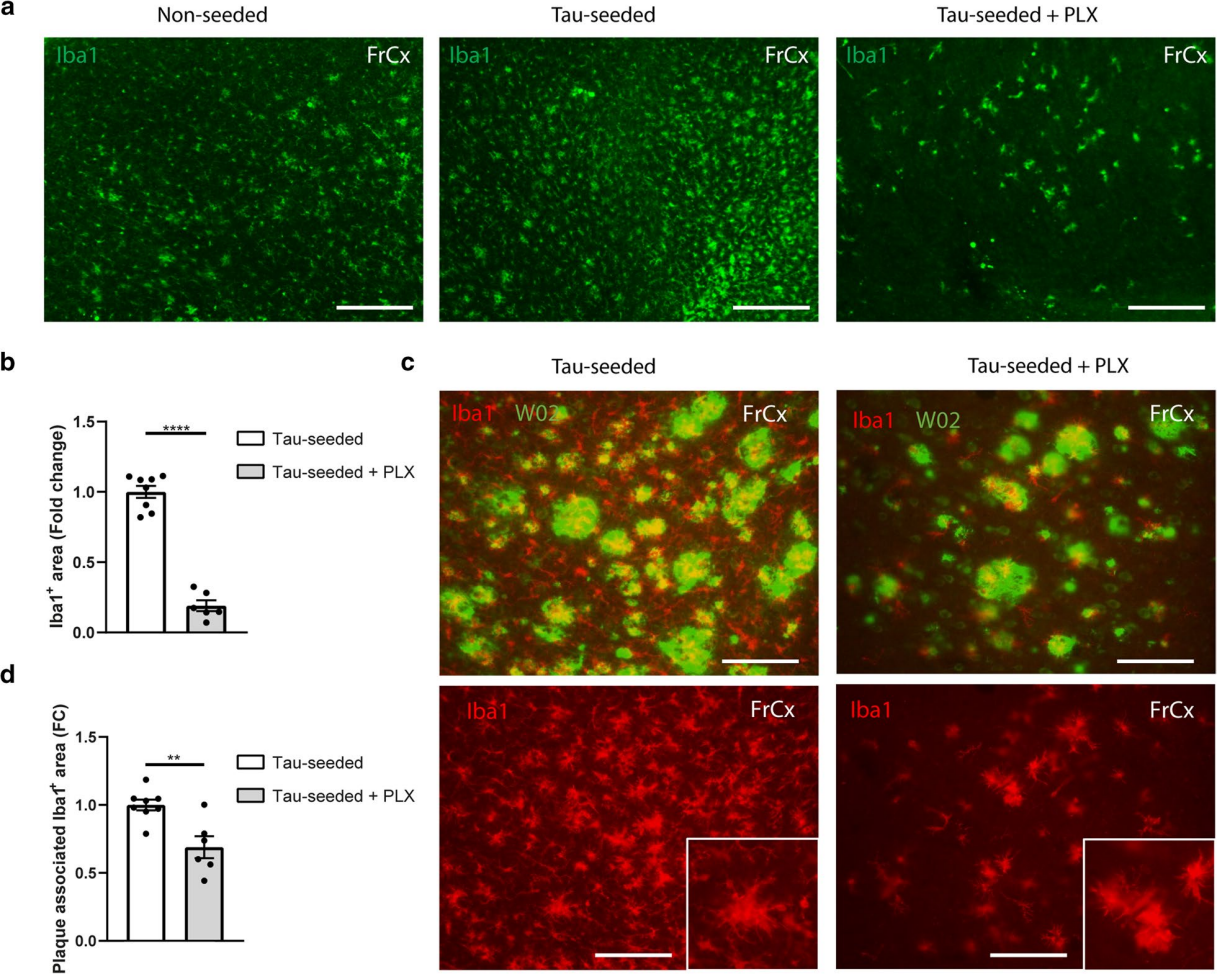
CSF1R inhibition preferentially depletes non-plaque associated microglia during ATN pathology. **a** Representative images of the frontal cortex of tau-seeded F^+^/T^+^ mice treated with control, or PLX3397 chow and PBS-injected littermates at 7 months of age (3 months post-injection), immunohistochemically stained with anti-Iba1 antibody. Scale bar = 250 µm. **b** Quantitative analysis of Iba1 signal in the cortex of PLX3397 treated tau-seeded F^+^/T^+^ mice (n = 6) compared to non-treated tau-seeded F^+^/T^+^ mice (n = 8). Unpaired t-test. **c** Representative images of the frontal cortex of tau-seeded F^+^/T^+^ mice treated with control or PLX3397 immunohistochemically stained with anti-Aβ antibody W02 and anti-Iba1 antibody and their respective overlay, demonstrating that remaining microglia after PLX3397 treatment reside in the vicinity of amyloid plaques. Scale bar = 100 µm. FrCx = frontal cortex. **d** Quantitative analysis of Iba1 signal associated with W02^+^-plaques in the frontal cortex of tau-seeded F^+^/T^+^ mice treated PLX3397 (n = 6), non-treated tau-seeded F^+^/T^+^ mice (n = 8). Plaque-associated Iba1 signal was quantified as the colocalizing signal and Iba1^+^ signal immediately bordering W02 positive area. Unpaired t-test. Data are presented as mean ± SEM; **p < 0.01; ****p < 0.0001

To further assess the contributory role of microglia under conditions of ATN pathology, we relied on microglial elimination via PLX3397 administration, a well-characterized CSF1R inhibitor [10]. PLX3397 was mixed in the chow at a concentration of 1 g/kg and administered to mice starting from 1.5 months post tau-seeding and maintained for 1.5 months. With this regimen we observed 81 ± 6% reduction of Iba1 ^+^ microglial area in the cortex (Fig. 6a, b). Remarkably, microglia that were not depleted following PLX3397 treatment displayed an activated morphology and were mostly associated with amyloid plaques (Fig. 6c), suggesting an increased resistance of plaque-associated microglia to CSF1R inhibition. Quantification of the plaque-associated Iba1 ^+^ area indicated 31 ± 8% reduction upon PLX treatment (Fig. 6d).

**Fig. 7:**
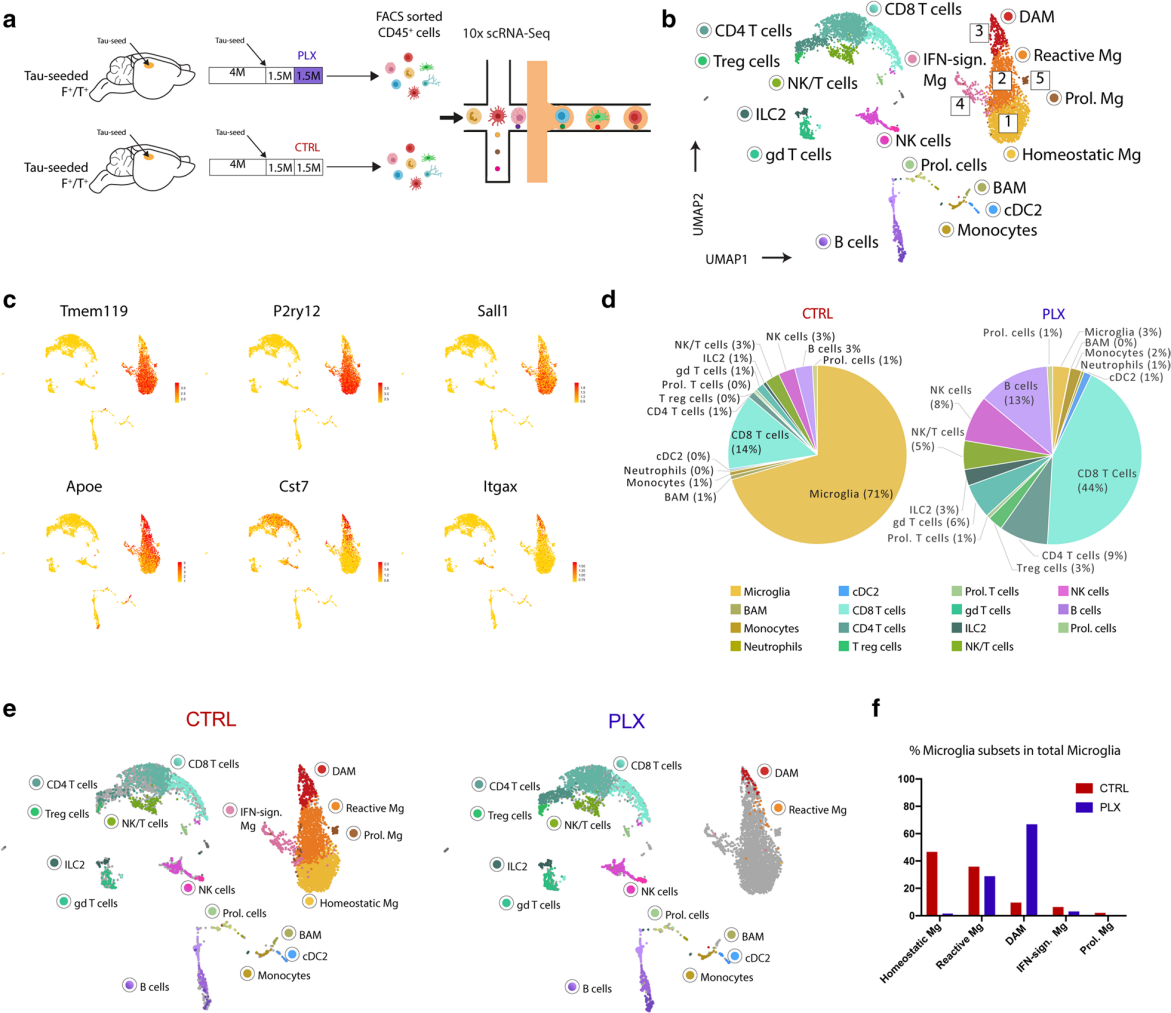
scRNA-Seq analysis of tau-seeded F^+^/T^+^ brains upon PLX3397 treatment. **a** Schematic overview of scRNA-Seq analysis in tau-seeded F^+^/T^+^ mice that were or were not treated with PLX3397 for 1.5 months following tau-seeding. Brains were collected from n = 2 mice for each condition. **b** UMAP-projection containing 4102 CD45^+^ cells originating from tau-seeded F^+^/T^+^ mice which had received control chow following tau-seeding, and 2130 CD45^+^ cells originating from tau-seeded F^+^/T^+^ mice which had received PLX chow following tau-seeding to deplete microglia. Clusters 1 to 5 correspond to different activation states of microglia population. **c** UMAP plots showing the expression of homeostatic microglia marker genes (top) and DAM marker genes (bottom). **d** Pie charts showing the percentages of each of the CD45^+^ subsets within the total CD45^+^ fraction in control (left) and PLX3397 treated (right) tau-seeded F^+^/T^+^ brains. **e** UMAP plots of the merged dataset of the control treated (left) and PLX3397 treated (right) tau-seeded F^+^/T^+^ whole brains as shown in **b**, where each UMAP plot highlights the cells from each condition separately. Clusters are colored according to the cluster colors shown in **b**. Only cells belonging to one of the 2 treatment conditions are colored per panel, cells belonging to the other treatment condition are depicted in grey. **f** Bar graphs showing the percentages of microglia subsets within total microglia per treatment group (red: control, blue: PLX)

The selective survival of plaque-associated microglia can be functionally relevant, as these cells may play an important role in plaque compaction and limiting toxicity [8, 68, 70, 73]. To further understand how PLX3397 treatment affects microglia under conditions of ATN pathology, we performed scRNA-Seq analysis on CD45 ^+^ immune cells isolated from brains of tau-seeded F^+^/T^+^ that were or were not treated with PLX chow for 1.5 months (Fig. 7a). 4102 immune cells from untreated brains and 2130 cells from PLX treated brains were pooled in a single dataset followed by UMAP projection and unsupervised clustering (Fig. 7b). In agreement with our previous results, a large fraction of microglia exhibited a clear activation signature (clusters 2, 3), with downregulation of homeostatic genes (*Tmem119*, *P2ry12*, *Sall1*) and an increase of DAM genes (*Apoe*, *Cst7*, *Itgax*) (Fig. 7c). Additionally, microglia with a proliferation (cluster 5) or an IFN-induced signature (cluster 4) were once again observed. PLX treatment clearly depleted microglia, as their percentage within the CD45 ^+^ brain immune compartment fell to 3% as compared to 71% in the untreated group (Fig. 7d). Importantly, nearly all remaining microglia in the PLX treated group exhibited a DAM or reactive phenotype (Fig. 7e, f). Other microglial subsets were nearly completely depleted in the PLX-treated mice (Fig. 7f). The remaining DAMs and reactive microglia in PLX treated brains showed an elevated expression of *Apoe*, as compared to their counterparts in untreated mice (Additional file 7: Supplementary Fig. 7). Together our data suggest that DAMs that associate with amyloid plaques are more resistant to CSF1R inhibition, with a significant fraction surviving high doses of PLX3397.

### CSF1R inhibition attenuates amyloid-facilitated tau pathology and neurodegeneration in tau-seeded 5xFAD/PS19 mice

**Fig. 8:**
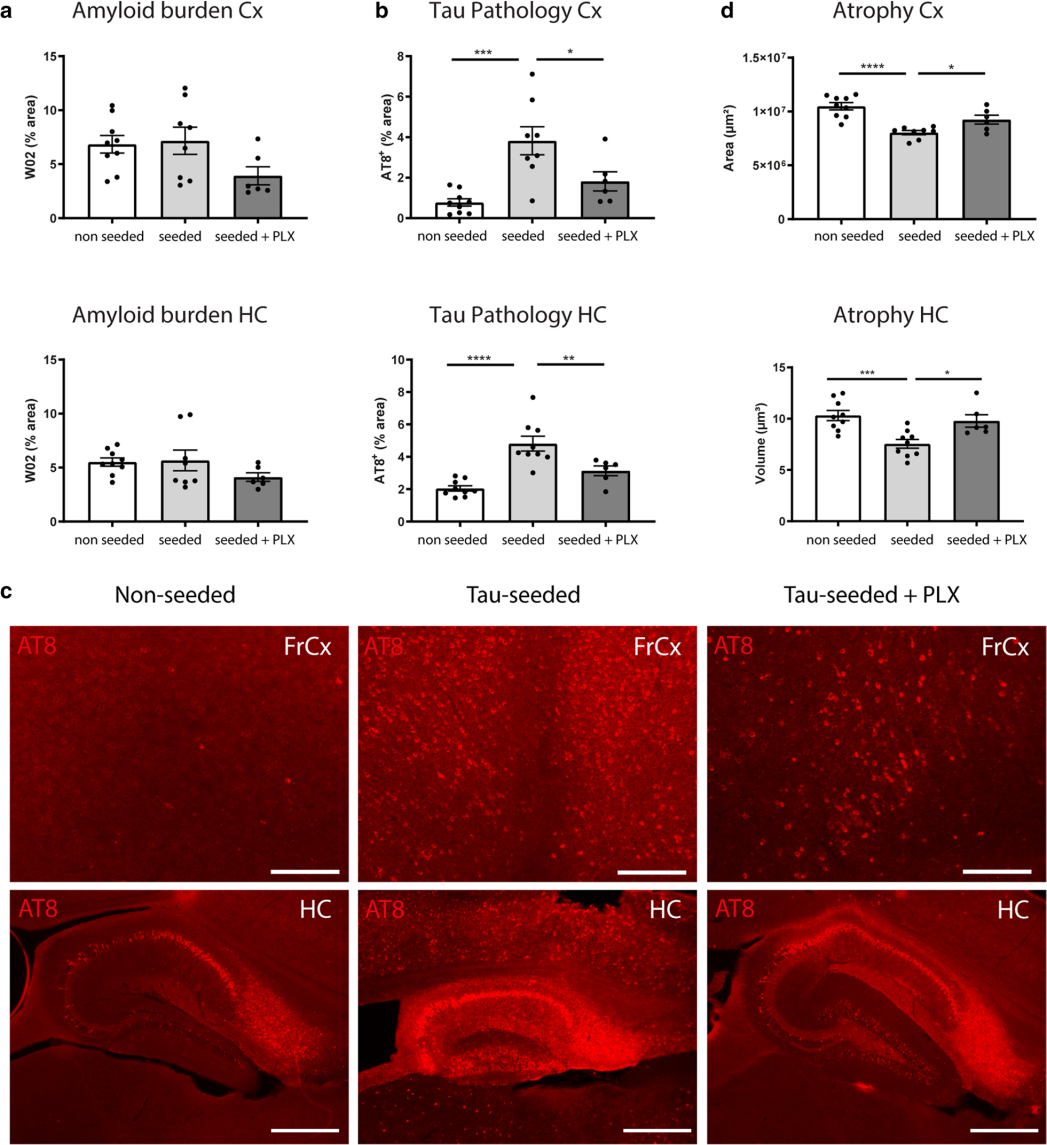
CSF1R inhibition attenuates amyloid-facilitated tau-seeded pathology and neurodegeneration. **a** Quantitative analysis of W02 signal in the cortex and hippocampus of PLX3397 treated tau-seeded F^+^/T^+^ mice (n = 6) compared to non-treated tau-seeded F^+^/T^+^ mice (n = 8) and non-seeded littermates (n = 9). **b** Quantitative analysis of AT8 signal in the cortex and hippocampus of PLX3397 treated tau-seeded F^+^/T^+^ mice (n = 6) compared to non-treated tau-seeded F^+^/T^+^ mice (n = 8 Cx, n = 9 HC) and non-seeded littermates (n = 9). Analyses were done at 7 months of age (3 months post-injection). Data are presented as mean ± SEM; *p < 0.05; **p < 0.01; ***p < 0.001 ****p < 0.0001. one-way ANOVA, Dunnett’s multiple comparison test. **c** Representative images of frontal cortex (scale bar = 250 µm) and hippocampus (scale bar = 500 µm) of PLX3397 treated tau-seeded F^+^/T^+^ mice, non-treated tau-seeded F^+^/T^+^ mice and non-seeded littermates at 7 months (3 months post-injection), immunohistochemically stained with anti-phospho-tau (pSer202/Thr205) antibody. **d** Quantification of cortical area and hippocampal volume in PLX3397 treated tau-seeded F^+^/T^+^ mice (n = 6) compared to non-treated tau-seeded F^+^/T^+^ mice (Cx n = 8, HC n = 9) and non-seeded littermates (n = 9). Data are presented as mean ± SEM; *p < 0.05; **p < 0.01; ***p < 0.001 ****p < 0.0001. one-way ANOVA, Dunnett’s multiple comparison test. FrCx = frontal cortex; HC = hippocampus

To assess the net effect of PLX3397 treatment and microglial depletion on ATN disease pathology, mice were analyzed after 1.5 months of treatment at 7 months of age. Immunohistochemical analysis revealed no significant change in amyloid pathology in the cortex or hippocampus (Fig. 8a). A tendency for decreased amyloid pathology was noted in cortex while not in hippocampus, displaying more mature plaques. The absence of significant inhibition in both regions is in line with previous work showing that microglial depletion does not affect mature plaque load, in contrast to early plaque development [55]. However, we observed that PLX3397 treatment significantly decreased tau pathology both in the cortex and hippocampus of tau-seeded mice (Fig. 8b, c). Importantly, microglial elimination also rescued cortical and hippocampal atrophy in tau-seeded mice, as the cortical area and hippocampal volume were significantly increased upon PLX3397 treatment (Fig. 8d). Our data show that in a model of amyloid-facilitated tau propagation and neuronal atrophy, CSF1R inhibition, which primarily depletes non-plaque-associated microglia, ameliorates tau pathology and neurodegeneration. This indicates that some microglial populations, targeted by CSF1R inhibition, exacerbate disease progression in the presence of combined ATN pathology.

## Discussion

The amyloid cascade hypothesis suggests a causal link between amyloid and tau pathology [20]. In human AD patients, the development of amyloid pathology is considered to be an early event that subsequently triggers and facilitates tau pathology and symptom progression. Mouse models support the notion of amyloid-facilitated tau pathology. Mouse lines that combine both amyloid and tau pathology have confirmed that amyloid pathology accelerates tau pathology [4, 6, 14, 15, 23, 33, 36, 45, 57]. Recent work has also shown that amyloid plaques facilitate tau-seeding, thereby enhancing aggregation of endogenous mouse tau upon seeding with human AD tau filaments [21, 66]. A model put forward by He et al. thereby showed that dystrophic neurites near amyloid plaques precede and may function as the initial hub for tau misfolding and aggregation [21]. Endogenous tau would accumulate within amyloid-associated dystrophic axons, and subsequently the formation of NP tau is triggered. The recruited tau seeds may thereby translocate to neuronal somas where they, at a slower rate, develop into NFTs. This represents a compelling model for amyloid-facilitated progressive tau pathology as observed in AD patients, and emphasizes the importance of this early process in AD. However, in this model no progression to robust atrophy was observed. In order to facilitate secondary effects and to boost amyloid-facilitated tau pathology beyond thresholds sufficient for neurodegeneration, we performed tau-seeding in tau transgenic mice in the presence of amyloid plaques to mimic ATN pathology. Our results now confirm that amyloid plaques strongly accelerate tau pathology in our tau-seeding model, which also progressed towards robust neuronal atrophy. The tau pathology we observed manifested itself as NFTs in neuronal somas. This suggests that under conditions of human mutant tau overexpression, NFT formation is strongly accelerated and overtakes NP tau. The use of mutant tau overexpression and tau-seeding presents concomitantly the limitation and the strength of our model. While this renders the model more artificial, it enables the generation of a setting that recapitulates combined ATN pathology, enabling analysis of amyloid-facilitated tau pathology and accelerated neurodegeneration. The efficient development of tau pathology in tau-seeded 5xFAD/PS19 mice likely explains the high level of atrophy we observed, while synergistic effects of amyloid and tau pathology must also be considered, in line with previously published models [36, 45]. We report a significant correlation between the level of tau pathology and hippocampal and cortical brain atrophy. This is in line with findings in AD patients where NFTs also correlate with symptom progression and atrophy [7, 19, 47]. While tau aggregation correlates with the neurodegenerative process, tau forms ranging from small oligomers to larger aggregates, may be the toxic culprit [13]. Several mechanisms may contribute to amyloid-facilitated seeding and propagation of tau-pathology [21, 59]. These include alterations of tau seed and/or tau acceptor properties, changes in tau post-translational modifications and alterations of seed homeostasis (clearance-uptake routes). And Aβ-induced-neuroinflammation, including microglial activation, may also exacerbate tau aggregation and/or propagation [59].

Neuroinflammation has been increasingly implicated in the pathogenesis of AD. Microglia are the key-sensors in the brain and react to danger signals that arise during AD progression [48]. Hence microglial profiles may be differentially modulated along the various stages of AD and may require distinct therapeutic approaches pending on the disease stage. Most importantly, fine-tuned therapeutic approaches targeting detrimental microglial populations, while conserving protective populations will need to be defined at different stages of the disease process, or ATN axis. Single-cell analysis first revealed the existence of DAMs in mouse amyloid models [29, 30, 65]. However, induction of DAMs is not restricted to amyloid pathology, as these cells are also observed in tau models of AD and exhibited a comparable gene expression profile as compared to DAMs from amyloid models [51]. DAMs are furthermore identified in models with neurodegeneration. Our results now reveal how microglia respond to settings of ATN pathology, showing an increased activation and transformation towards DAMs as compared to amyloid-only models. This is likely a result of the high levels of neurodegeneration and cell death, which is a known driver of the DAM state in microglia [30]. We also observed an increase in *Apoe* expression globally across all microglia in our ATN model. Upregulation of *Apoe* in microglia may contribute to their subsequent transformation towards DAMs, as this has been shown to rely on TREM2-APOE-mediated signaling [30]. *APOE* expression is also increased in microglia from human AD patients, as observed via snRNA-Seq [18, 40, 74]. The upregulation of APOE may have important functional consequences. ApoE may independently affect amyloid and tau pathology, while it can also be involved in the link between both pathologies. APOE may contribute to tau propagation, as its deletion rescues tau pathology and neurodegeneration in tau transgenic mice [50]. Importantly, APOE-dependent pathology was shown to be driven by microglia [49]. Furthermore, knock-in of the human *APOE4* variant exacerbates tau pathology and neurodegeneration as compared to *APOE2* and *APOE3*. In humans, *APOE4* carriers display a higher level of neurodegeneration for a similar level of amyloid pathology [11, 63] and seem to progress faster to more advanced stages of the disease [67]. A PET-study in humans also demonstrated a combined effect of *APOE* status and amyloid load on tau pathology and spreading [61]. Importantly, recent work identified a protective APOE mutation in a human autosomal dominant AD setting. An *APOE3* Christchurch (R136S) mutation carrier did not develop strong tau pathology and neurodegeneration and remained symptom free until late age [1]. In the same vein, the *APOE2* allele is associated with a lower risk of AD dementia [46]. In conclusion, a central disease-modifying role for APOE is emerging. The increased microglial production of *Apoe* upon ATN settings may exacerbate tau pathology and neurodegeneration.

Microglia may differentially affect disease progression at different stages along the ATN axis. Until now most preclinical studies have assessed their contribution either in an amyloid or tau model of AD. As stated in the introduction at early stages of amyloid pathology, CSF1R inhibition decreases amyloid plaque pathology and also decreases early forms of accumulating amyloid including intraneuronal and soluble Aβ forms, and Aβ oligomers [32, 53–55]. These are also considered important contributors of cognitive and synaptic dysfunction in AD and preclinical AD models [32, 41]. However, it remained unclear how microglia would modulate disease progression in a combined ATN setting, which mimics human AD progression. To assess the net effect of microglia on disease progression in our ATN model, we attempted to deplete them via CSF1R inhibition. While CSF1R inhibition is a well accepted approach to assess the role of microglia in neurodegenerative diseases [17], potential side-effects must be considered, as well as potential contributing roles of perivascular and meningeal macrophages, which are also eliminated. However the use of CSF1R inhibitors provides a versatile approach enabling the elimination of microglia at a chosen time-point. We have chosen a time point for CSF1R inhibition, characterized by mature plaque pathology in our cohort and after initiation of tau-seeding, to assess the effect on amyloid facilitated tau-seeding and subsequent neurodegeneration. Importantly, while non-plaque associated microglia were efficiently depleted, plaque-associated microglia were partially resistant. Previous work has also shown that in amyloid-only models, plaque-associated microglia were more resistant to CSF1R inhibition [55, 64]. By relying on scRNA-Seq analysis we now reveal that the surviving plaque-associated microglia were DAMs. As our data suggest that under ATN conditions not all DAMs associate with amyloid plaques, it is intriguing that primarily the plaque-associated DAMs were more resistant to PLX3397 treatment. DAMs that are in close contact to amyloid plaques may have active signaling pathways that provide a survival signal and thus render them less dependent on CSF1R signaling. A possible candidate is TREM2 signaling [69], which is likely more pronounced around amyloid plaques. A near complete depletion of non-plaque associated microglia and a less than two-fold reduction in plaque-associated DAMs significantly ameliorated tau pathology and neuronal atrophy. This reveals the detrimental contribution of microglia to disease pathology during ATN progression and is in line with previous work that has reported their detrimental role in tau models of AD [2, 16, 25, 31, 35, 38, 39, 56, 72]. However, it is possible that plaque-associated DAMs compact amyloid plaques and limit amyloid toxicity [8, 68, 70, 73]. While this needs to be further examined under conditions of ATN progression, their incomplete depletion may thus be an asset for therapeutic targeting. Our results highlight how CSF1R inhibition allows for depletion of microglial subsets, while selectively sparing other subsets, i.e. plaque associated microglia. Indeed, we envisage that a certain dosage of inhibitor may spare the majority of plaque-associated DAMs—which may potentially limit amyloid toxicity—while efficiently depleting non-plaque associated microglia populations that may exacerbate tau pathology. Taken together we here show that CSF1R inhibition rescues tau pathology and neurodegeneration, while preserving plaque associated microglia, in an ATN model, recapitulating amyloid-facilitated tau propagation and neurodegeneration. Microglia-directed treatments that specifically target detrimental populations, while sparing and/or promoting beneficial microglial responses may yield new therapeutic opportunities. Our work offers a framework that can be used in this endeavor.

## Supplementary Information

**Additional file 1: Fig. S1**. Progressive development of amyloid pathology in 5xFAD mice. Representative images of the frontal cortex and hippocampus of F^+^/T^−^ mice stained with W02 antibody showing the evolution of amyloid pathology in subiculum from 2.5 months to extension of amyloid pathology to the frontal cortex at 4 months and robust pathology at 7 months. Scale bar = 500 µm (FrCx = frontal cortex; HC = hippocampus). Quantitative analysis of W02 staining in frontal cortex and hippocampus of 2.5 months (n = 3), 4 months (n = 3) and 7 months (n = 3) old F^+^/T^−^ mice. Data are presented as mean ± SEM; *p < 0.05; **p < 0.01; ***p < 0.001 one-way ANOVA with Tukey’s multiple comparison test

**Additional file 2: Fig. S2**. Tau-seeding induces propagation of tau pathology to brain regions remote from the injection site. **a** AT8 staining of tau pathology was semi-quantitatively scored from 0-3 in different brain regions generating heat maps of in sagittal brain slices of 7 months old tau-seeded F^+^/T^+^ and F^−^/T^+^ mice and their non-seeded littermates. **b **Immunohistological staining of tau pathology with anti-phospho-tau (pSer202/Thr205) antibody AT8 on the brain stem and thalamus of tau-seeded F^−^/T^+^ and F^+^/T^+^ mice. Scale bar = 250 µm. Quantitative analysis of tau pathology (measured as AT8 stained area) in the ipsi-lateral brainstem and thalamus of tau-seeded F^−^/T^+^ and F^+^/T^+^ mice (n = 8; n = 6) compared to non-seeded F^−^/T^+^ and F^+^/T^+^ mice (n = 9; n = 9). Data are presented as mean ± SEM; ****p < 0.0001 two-way ANOVA, Tukey’s test for multiple comparison

**Additional file 3: Fig. S3**. Amyloid-pathology facilitates propagation of tau-seeded tau pathology and tau-induced atrophy. **a,b** Representative images of sagittal brain sections of 7 months old tau-seeded F^−^/T^+^ and F^+^/T^+^ in frontal cortex and hippocampus at 3 months post injection, and their non-seeded littermates, immunohistochemically stained with **(a) **anti-phospho-tau antibody AT8 and **(b)** anti-NeuN antibody. Scale bar = 2 mm

**Additional file 4: Fig. S4**. Amyloid-pathology aggravates tau-induced cortical atrophy. **a.** Representative images of the cortex of tau-seeded F^−^/T^+^ and F^+^/T^+^ mice and their non-seeded littermates at 7 months (3 months post-injection), immunohistochemically stained with anti-NeuN antibody. Scale bar = 500 µm. **b** Quantification of cortical area of tau-seeded F^+^/T^+^ mice (n = 6) compared to tau-seeded F^−^/T^+^ mice (n = 8) and non-seeded F^−^/T^+^ and F^+^/T^+^ mice (n = 9; n = 9). Two-way ANOVA, Tukey’s test for multiple comparison. **c** Correlation analysis between tau pathology in the cortex and cortical atrophy in 7 months old tau-seeded and non-seeded F^−^/T^+^ and F^+^/T^+^ mice. Pearson’s correlation analysis. **d** Quantitative analysis of cortical and hippocampal atrophy in the contra-lateral hemisphere of tau-seeded F^+^/T^+^ compared to tau-seeded F^−^/T^+^ mice (n = 6; n = 8) and non-seeded F^+^/T^+^ and F^−^/T^+^ mice (n = 9; n = 9). Two-way ANOVA, Tukey’s test for multiple comparison. **e** Quantitative analysis of inverted grid hanging of tau-seeded F^−^/T^+^ and F^+^/T^+^ mice (n = 8; n = 6) 3 months post-injection, as well as their non-seeded littermates (n = 9; n = 9). Two-way ANOVA, Tukey’s test for multiple comparison. Data are presented as mean ± SEM, **p < 0.01; ***p < 0.001; ****p < 0.0001

**Additional file 5: Fig. S5**. Microgliosis in the presence of amyloid pathology, tau pathology and combined ATN pathology. **a, b** Representative images of (**a**) frontal cortex (Scale bar = 250 µm) and (**b**) hippocampus** (**Scale bar = 500 µm) of wildtype F^−^/T^−^, non-seeded F^−^/T^+^, tau-seeded F^−^/T^+^, F^+^/T^−^ and tau-seeded F^+^/T^+^ mice at 7 months of age, immunohistochemically stained with anti-Iba1 antibody, anti-phospho-tau (pSer202/Thr205) antibody AT8 or anti-Aβ antibody W02. Quantitative analysis of Iba1 signal in F^−^/T^−^ (n = 6), F^−^/T^+^(n = 9), tau-seeded F^−^/T^+^ (n = 8), F^+^/T^−^ (n = 6) and tau-seeded F^+^/T^+^ (n = 6) mice. One-way ANOVA with Tukey’s multiple comparison test. Data are presented as mean ± SEM; *p < 0.05; **p < 0.01; ****p < 0.0001

**Additional file 6: Fig. S6**. ATN pathology increases general and microglia-related expression of ApoE. **a, b** Representative images of frontal cortex of F^−^/T^−^, F^+^/T^−^ and tau-seeded F^+^/T^+^ mice at 7 months of age, immunohistochemically stained with anti-ApoE antibody, anti-Aβ antibody W02, and **(a)** anti-CD68 antibody or **(b)** anti-Iba1 antibody. Scale bar = 100 µm. Quantitative analysis of total ApoE staining and ApoE staining in microglia in F^−^/T^−^ (n = 6), F^+^/T^−^ (n = 6) and tau-seeded F^+^/T^+^ (n = 6) mice. One-way ANOVA with Tukey’s multiple comparison test. Data are presented as mean ± SEM; *p < 0.05; **p < 0.01; ***p < 0.001; ****p < 0.0001** c **Representative images of the frontal cortex of tau-seeded F^+^/T^+^ mice at 7 months of age, immunohistochemically stained with anti-ApoE antibody, anti-Aβ antibody W02, and anti-Iba1 antibody showing non-plaque associated microglia containing ApoE (white arrows). Scale bar = 25 µm

**Additional file 7: Fig. S7**. *Apoe* expression in PLX-treated versus control-treated reactive microglia and DAM isolated from whole brains of tau-seeded F^+^/T^+^ mice. Violin plots showing the normalized gene expression of *Apoe* per cell in reactive microglia, and DAM isolated from tau-seeded F^+^/T^+^ mouse models which had received PLX treatment (blue) or control treatment (red)
