# Supplementary figures and images for: CSF1R inhibition rescues tau pathology and neurodegeneration in an A/T/N model with combined AD pathologies, while preserving plaque associated microglia
Source: Acta Neuropathol Commun. 2021 Jun 8;9:108. doi: 10.1186/s40478-021-01204-8 (PMC8188790; doi:10.1186/s40478-021-01204-8)

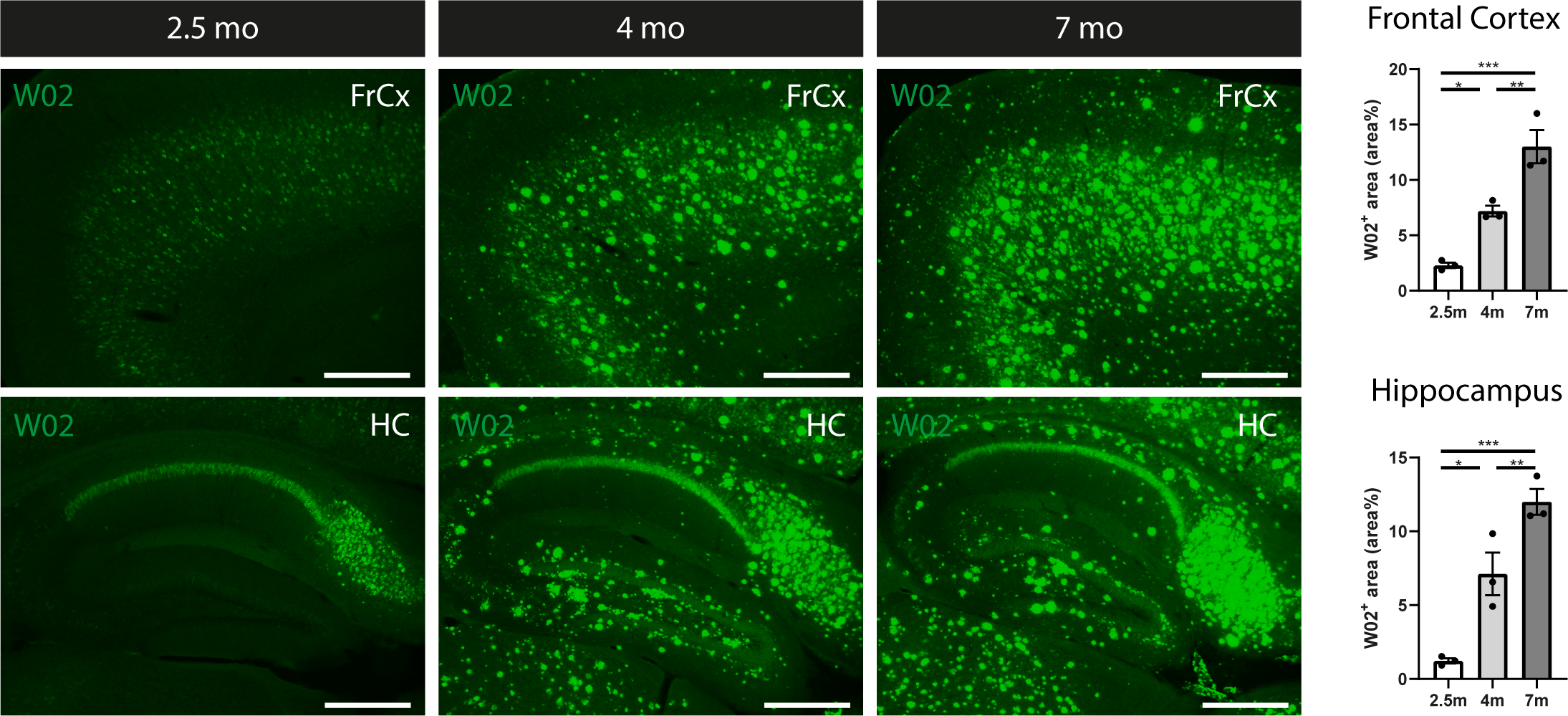

Supplement: Supplementary file 1 — Additional file 1: Fig. S1. Progressive development of amyloid pathology in 5xFAD mice. Representative images of the frontal cortex and hippocampus of F+/T− mice stained with W02 antibody showing the evolution of amyloid pathology in subiculum from 2.5 months to extension of amyloid pathology to the frontal cortex at 4 months and robust pathology at 7 months. Scale bar = 500 µm (FrCx = frontal cortex; HC = hippocampus). Quantitative analysis of W02 staining in frontal cortex and hippocampus of 2.5 months (n = 3), 4 months (n = 3) and 7 months (n = 3) old F+/T− mice. Data are presented as mean ± SEM; *p < 0.05; **p < 0.01; ***p < 0.001 one-way ANOVA with Tukey’s multiple comparison test [file 40478_2021_1204_MOESM1_ESM.tif]

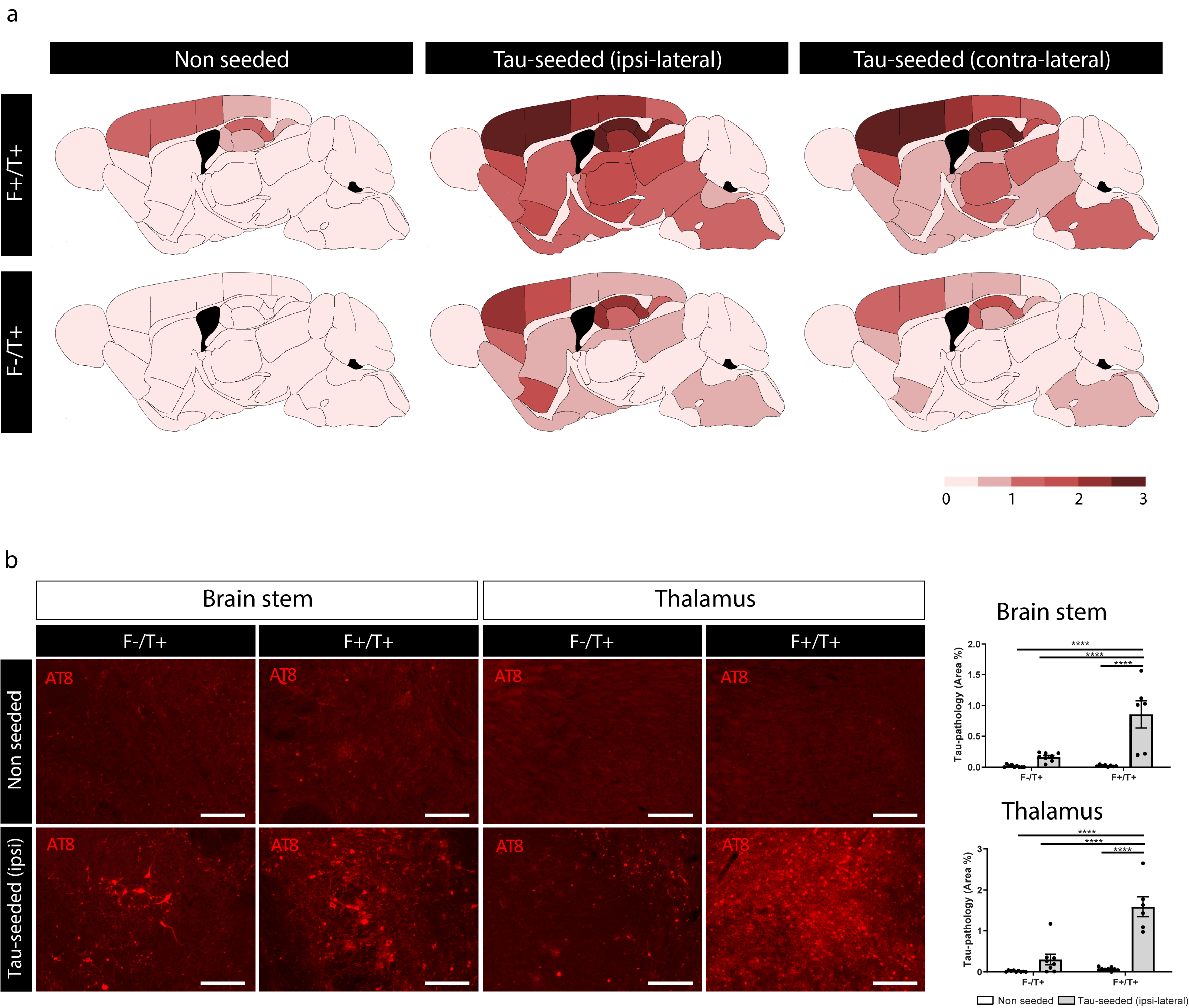

Supplement: Supplementary file 2 — Additional file 2: Fig. S2. Tau-seeding induces propagation of tau pathology to brain regions remote from the injection site. a AT8 staining of tau pathology was semi-quantitatively scored from 0-3 in different brain regions generating heat maps of in sagittal brain slices of 7 months old tau-seeded F+/T+ and F−/T+ mice and their non-seeded littermates. b Immunohistological staining of tau pathology with anti-phospho-tau (pSer202/Thr205) antibody AT8 on the brain stem and thalamus of tau-seeded F−/T+ and F+/T+ mice. Scale bar = 250 µm. Quantitative analysis of tau pathology (measured as AT8 stained area) in the ipsi-lateral brainstem and thalamus of tau-seeded F−/T+ and F+/T+ mice (n = 8; n = 6) compared to non-seeded F−/T+ and F+/T+ mice (n = 9; n = 9). Data are presented as mean ± SEM; ****p < 0.0001 two-way ANOVA, Tukey’s test for multiple comparison [file 40478_2021_1204_MOESM2_ESM.tif]

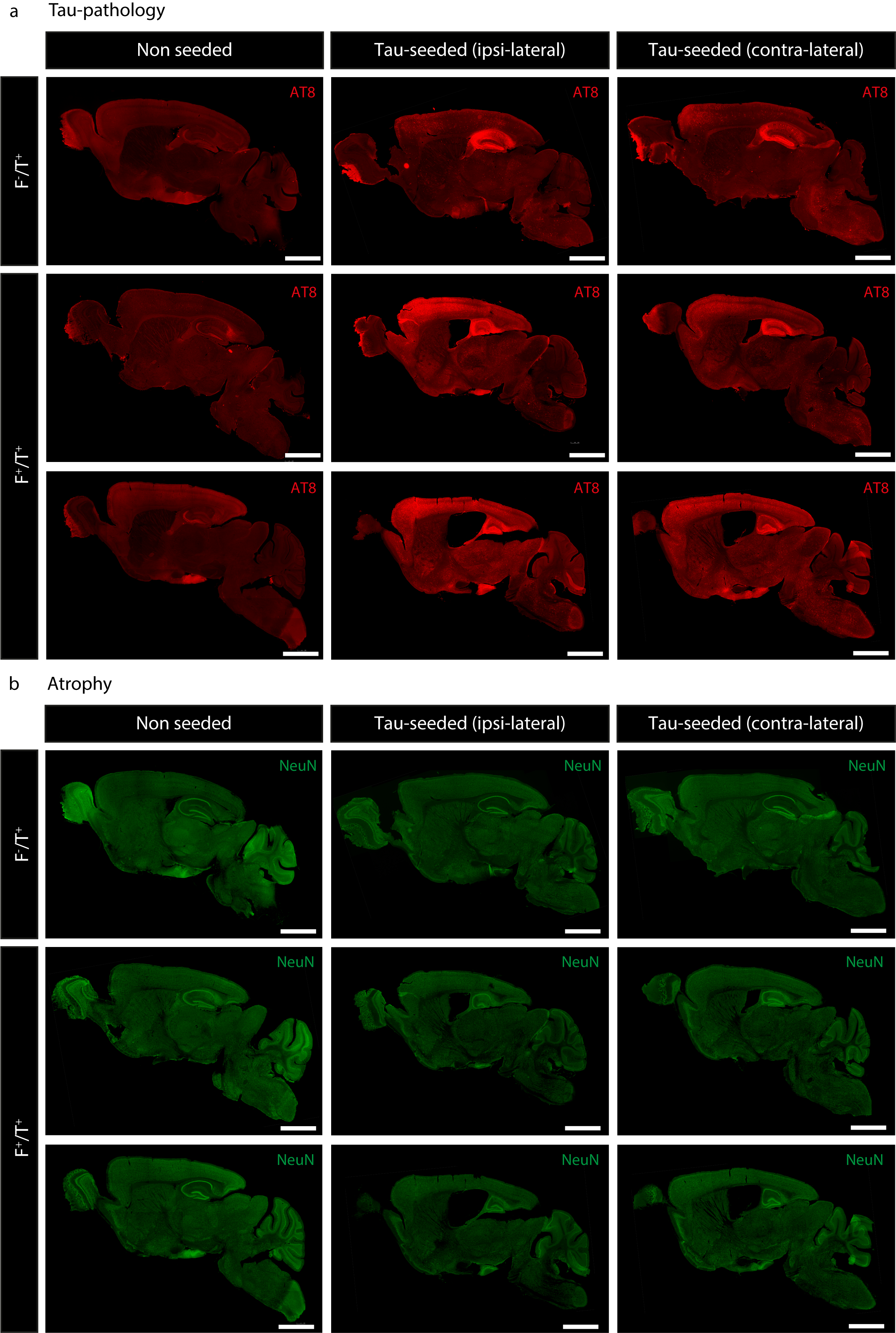

Supplement: Supplementary file 3 — Additional file 3: Fig. S3. Amyloid-pathology facilitates propagation of tau-seeded tau pathology and tau-induced atrophy. a,b Representative images of sagittal brain sections of 7 months old tau-seeded F−/T+ and F+/T+ in frontal cortex and hippocampus at 3 months post injection, and their non-seeded littermates, immunohistochemically stained with (a) anti-phospho-tau antibody AT8 and (b) anti-NeuN antibody. Scale bar = 2 mm [file 40478_2021_1204_MOESM3_ESM.tif]

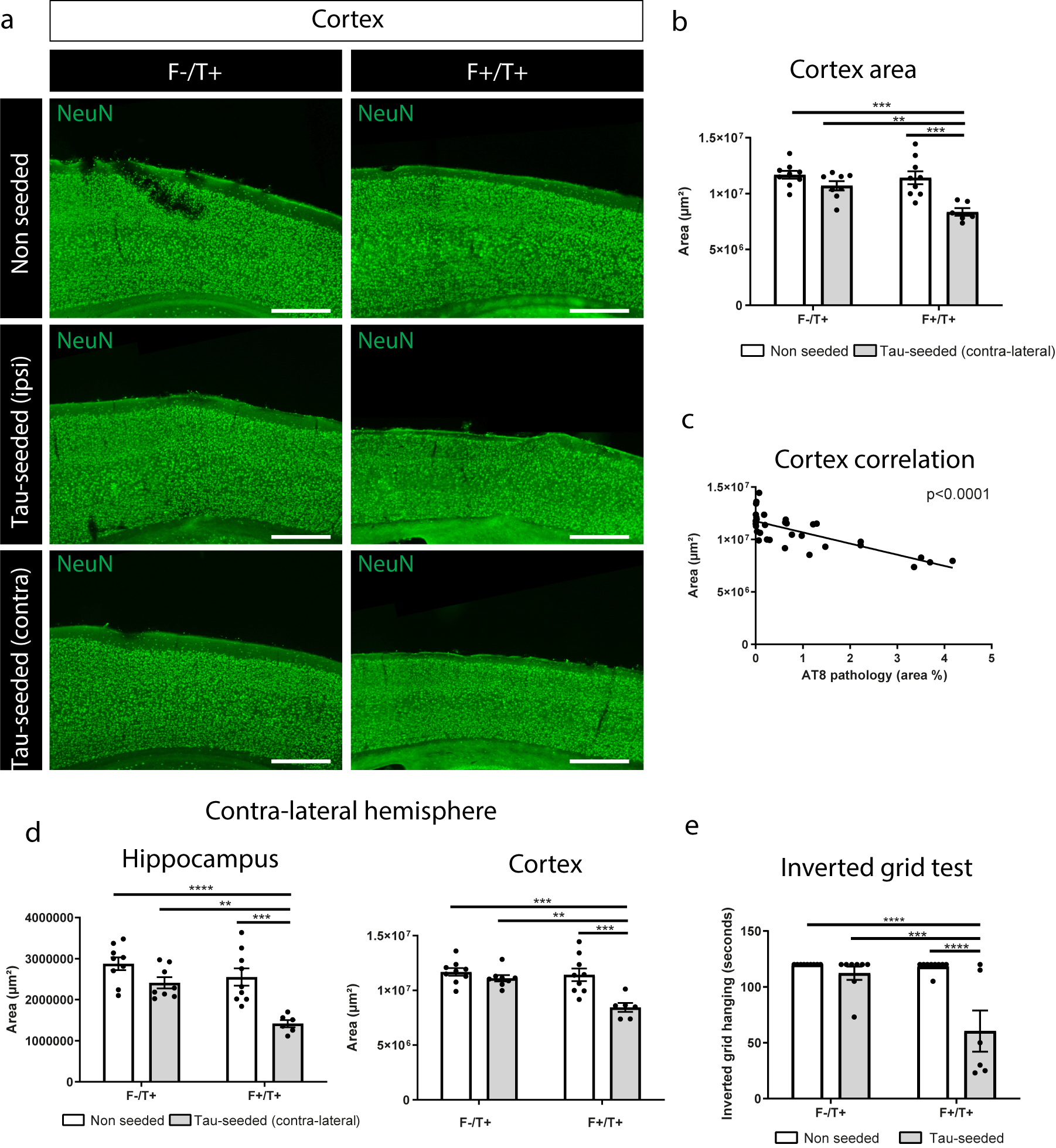

Supplement: Supplementary file 4 — Additional file 4: Fig. S4. Amyloid-pathology aggravates tau-induced cortical atrophy. a. Representative images of the cortex of tau-seeded F−/T+ and F+/T+ mice and their non-seeded littermates at 7 months (3 months post-injection), immunohistochemically stained with anti-NeuN antibody. Scale bar = 500 µm. b Quantification of cortical area of tau-seeded F+/T+ mice (n = 6) compared to tau-seeded F−/T+ mice (n = 8) and non-seeded F−/T+ and F+/T+ mice (n = 9; n = 9). Two-way ANOVA, Tukey’s test for multiple comparison. c Correlation analysis between tau pathology in the cortex and cortical atrophy in 7 months old tau-seeded and non-seeded F−/T+ and F+/T+ mice. Pearson’s correlation analysis. d Quantitative analysis of cortical and hippocampal atrophy in the contra-lateral hemisphere of tau-seeded F+/T+ compared to tau-seeded F−/T+ mice (n = 6; n = 8) and non-seeded F+/T+ and F−/T+ mice (n = 9; n = 9). Two-way ANOVA, Tukey’s test for multiple comparison. e Quantitative analysis of inverted grid hanging of tau-seeded F−/T+ and F+/T+ mice (n = 8; n = 6) 3 months post-injection, as well as their non-seeded littermates (n = 9; n = 9). Two-way ANOVA, Tukey’s test for multiple comparison. Data are presented as mean ± SEM, **p < 0.01; ***p < 0.001; ****p < 0.0001 [file 40478_2021_1204_MOESM4_ESM.tif]

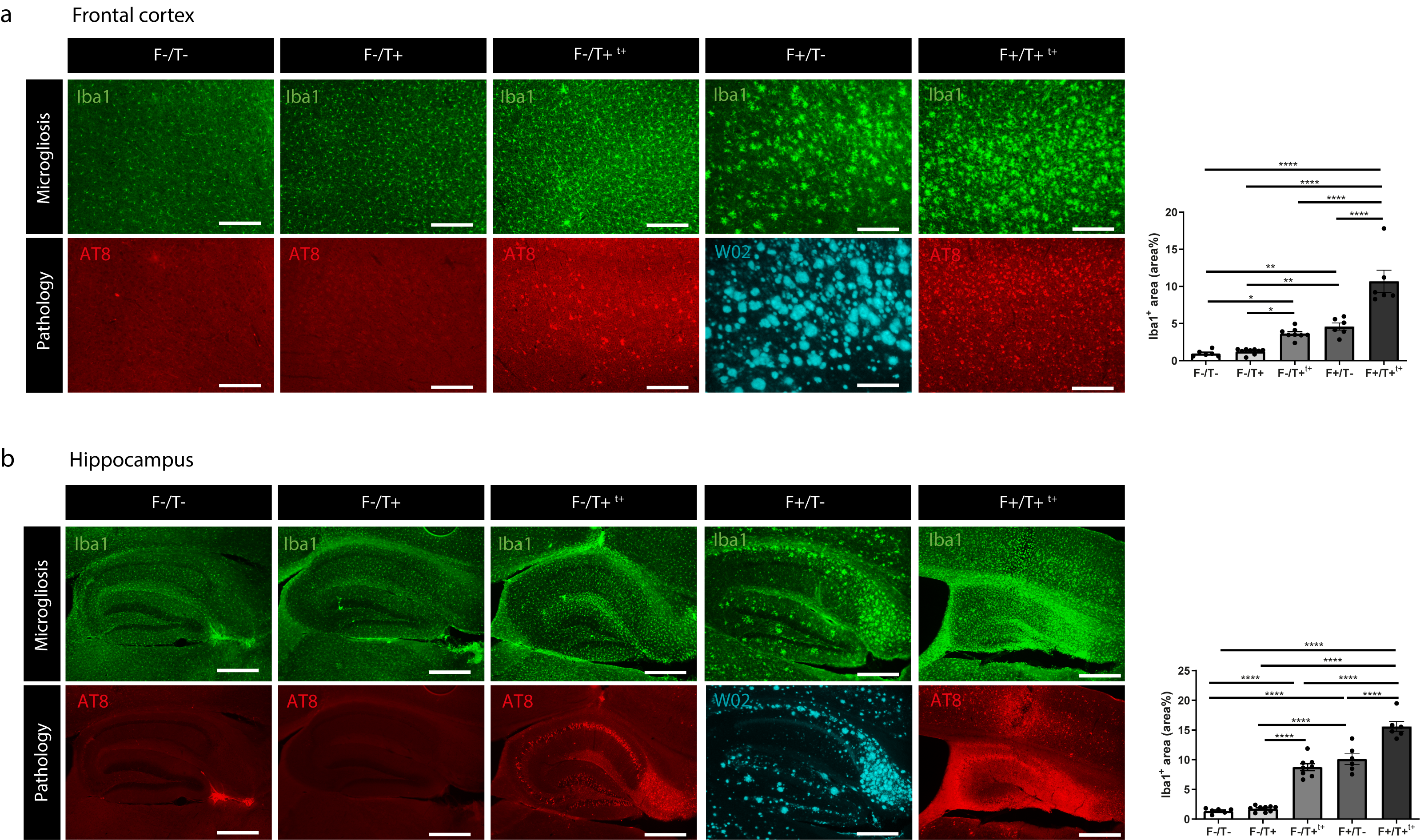

Supplement: Supplementary file 5 — Additional file 5: Fig. S5. Microgliosis in the presence of amyloid pathology, tau pathology and combined ATN pathology. a, b Representative images of (a) frontal cortex (Scale bar = 250 µm) and (b) hippocampus (Scale bar = 500 µm) of wildtype F−/T−, non-seeded F−/T+, tau-seeded F−/T+, F+/T− and tau-seeded F+/T+ mice at 7 months of age, immunohistochemically stained with anti-Iba1 antibody, anti-phospho-tau (pSer202/Thr205) antibody AT8 or anti-Aβ antibody W02. Quantitative analysis of Iba1 signal in F−/T− (n = 6), F−/T+(n = 9), tau-seeded F−/T+ (n = 8), F+/T− (n = 6) and tau-seeded F+/T+ (n = 6) mice. One-way ANOVA with Tukey’s multiple comparison test. Data are presented as mean ± SEM; *p < 0.05; **p < 0.01; ****p < 0.0001 [file 40478_2021_1204_MOESM5_ESM.tif]

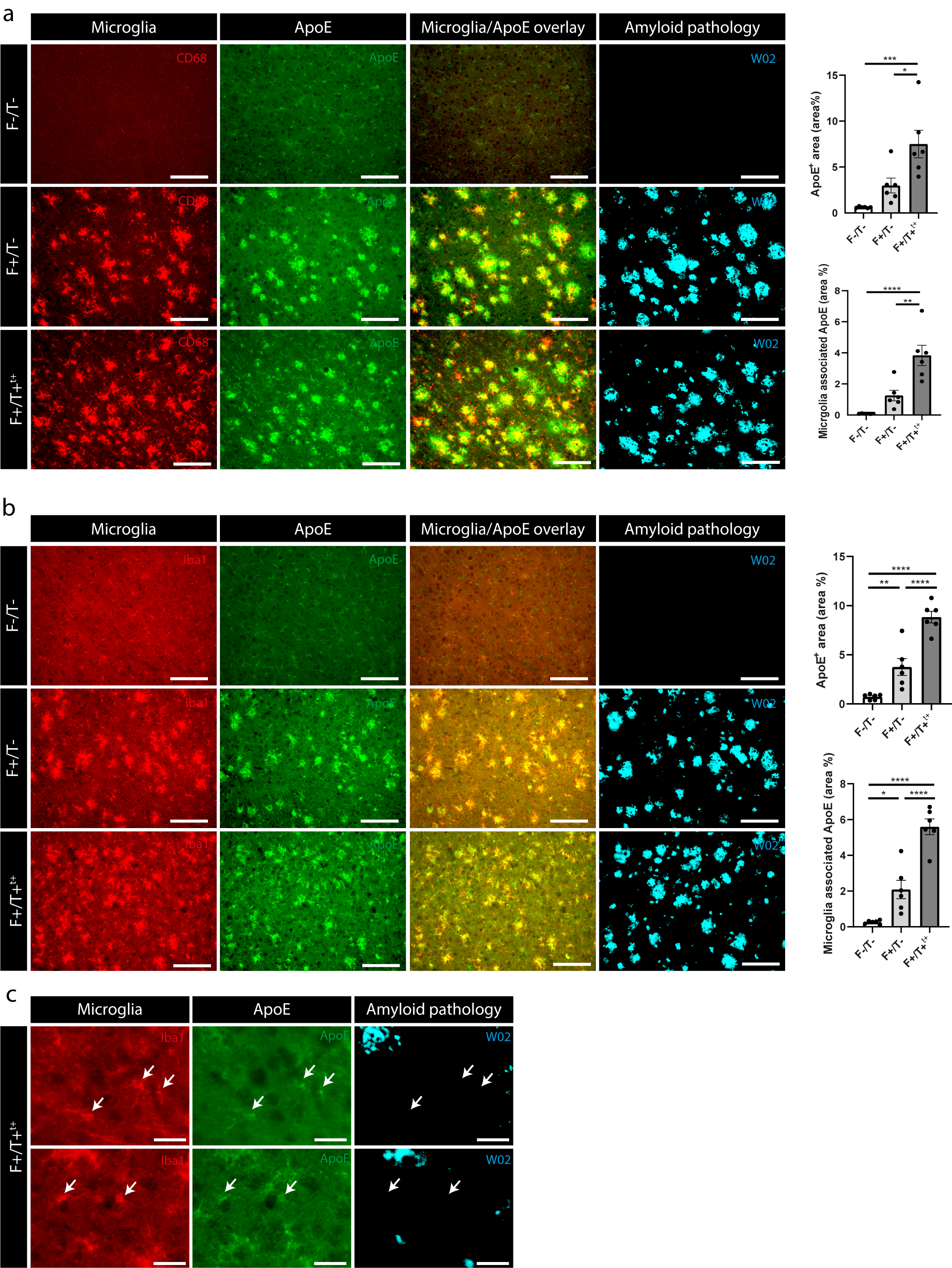

Supplement: Supplementary file 6 — Additional file 6: Fig. S6. ATN pathology increases general and microglia-related expression of ApoE. a, b Representative images of frontal cortex of F−/T−, F+/T− and tau-seeded F+/T+ mice at 7 months of age, immunohistochemically stained with anti-ApoE antibody, anti-Aβ antibody W02, and (a) anti-CD68 antibody or (b) anti-Iba1 antibody. Scale bar = 100 µm. Quantitative analysis of total ApoE staining and ApoE staining in microglia in F−/T− (n = 6), F+/T− (n = 6) and tau-seeded F+/T+ (n = 6) mice. One-way ANOVA with Tukey’s multiple comparison test. Data are presented as mean ± SEM; *p < 0.05; **p < 0.01; ***p < 0.001; ****p < 0.0001 c Representative images of the frontal cortex of tau-seeded F+/T+ mice at 7 months of age, immunohistochemically stained with anti-ApoE antibody, anti-Aβ antibody W02, and anti-Iba1 antibody showing non-plaque associated microglia containing ApoE (white arrows). Scale bar = 25 µm [file 40478_2021_1204_MOESM6_ESM.tif]

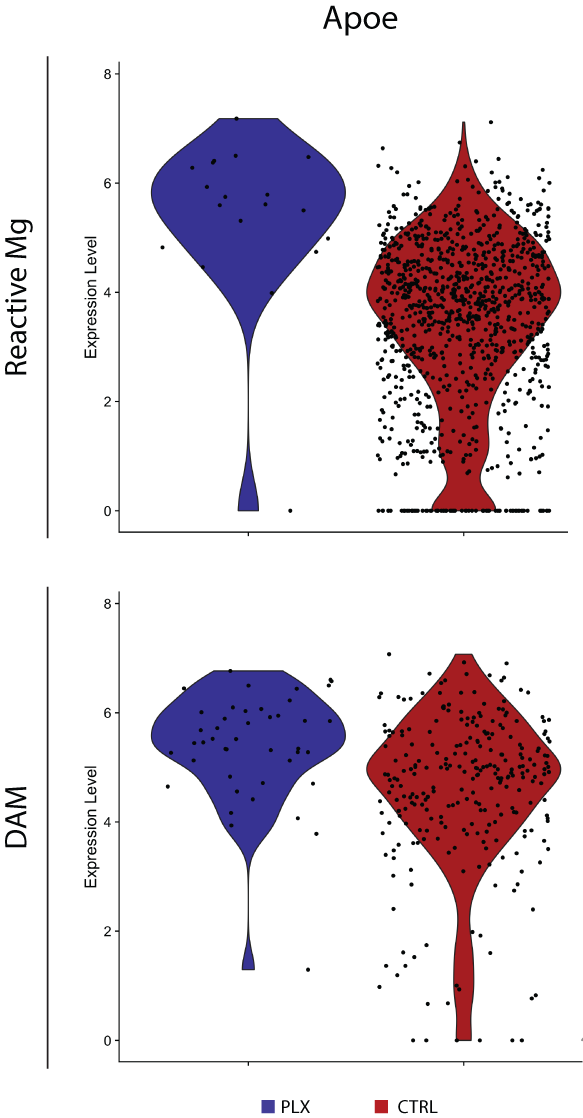

Supplement: Supplementary file 7 — Additional file 7: Fig. S7. Apoe expression in PLX-treated versus control-treated reactive microglia and DAM isolated from whole brains of tau-seeded F+/T+ mice. Violin plots showing the normalized gene expression of Apoe per cell in reactive microglia, and DAM isolated from tau-seeded F+/T+ mouse models which had received PLX treatment (blue) or control treatment (red) [file 40478_2021_1204_MOESM7_ESM.tif]
